# A glial perspective on the extracellular matrix and perineuronal net remodeling in the central nervous system

**DOI:** 10.3389/fncel.2022.1022754

**Published:** 2022-10-20

**Authors:** Bhanu P. Tewari, Lata Chaunsali, Courtney E. Prim, Harald Sontheimer

**Affiliations:** Department of Neuroscience, Center for Brain Immunology and Glia, School of Medicine, University of Virginia, Charlottesville, VA, United States

**Keywords:** perineuronal nets (PNNs), astrocytes, extracellular matrix (ECM), microglia, PV neurons, matrix metalloproteinases

## Abstract

A structural scaffold embedding brain cells and vasculature is known as extracellular matrix (ECM). The physical appearance of ECM in the central nervous system (CNS) ranges from a diffused, homogeneous, amorphous, and nearly omnipresent matrix to highly organized distinct morphologies such as basement membranes and perineuronal nets (PNNs). ECM changes its composition and organization during development, adulthood, aging, and in several CNS pathologies. This spatiotemporal dynamic nature of the ECM and PNNs brings a unique versatility to their functions spanning from neurogenesis, cell migration and differentiation, axonal growth, and pathfinding cues, etc., in the developing brain, to stabilizing synapses, neuromodulation, and being an active partner of tetrapartite synapses in the adult brain. The malleability of ECM and PNNs is governed by both intrinsic and extrinsic factors. Glial cells are among the major extrinsic factors that facilitate the remodeling of ECM and PNN, thereby acting as key regulators of diverse functions of ECM and PNN in health and diseases. In this review, we discuss recent advances in our understanding of PNNs and how glial cells are central to ECM and PNN remodeling in normal and pathological states of the CNS.

## Introduction

In 1895, Hens Gierke proposed the idea of a homogeneous and amorphous ground substance that embeds neuroglia and forms the structural architecture of the brain (Celio, 1999). Soon after, in 1898 Camillo Golgi described a pericellular coating around specific neurons in his seminal study on the eponymous Golgi complex (Celio et al., 1998; Celio, 1999). After nearly a century of relative obscurity, these structures unambiguously established themselves as different forms of neuroglial-embedding extracellular matrix (ECM) known as interstitial matrix and perineuronal nets (PNNs). Brain ECM is rich in hyaluronan (HA), chondroitin sulfate proteoglycans (CSPGs), and glycoproteins, with a minor proportion of fibrous proteins. Together in conjunction with water, ions, and secreted molecules, ECM creates a functionally dynamic extracellular milieu that provides structural support and effectuates diverse neuromodulatory functions (Hrabetova et al., 2018; Fawcett et al., 2019).

A large fraction of ECM is homogeneous and amorphous; however, several morphologically distinct forms are distributed throughout the brain (Fawcett et al., 2019; Patel et al., 2019; Chaunsali et al., 2021). A thin sheet-like condensation of ECM molecules on the pial surface and around parenchymal vasculature forms basement membranes (BMs) which carries out structural, signaling, and barrier functions. Another phenotypic specialization of ECM is PNN, which is a lattice-like condensation predominantly juxtaposing the soma, dendrites, and axon initial segment (AIS). A vast majority of PNN-expressing neurons are fast-spiking parvalbumin (PV)-expressing GABAergic neurons; however, several non-PV neurons also express PNNs (Lensjø et al., 2017a; Patel et al., 2019; Chaunsali et al., 2021). Brain ECM, including PNNs, is spatiotemporally malleable and maintains a characteristic composition and structural organization at different stages of pre and postnatal development, adulthood, aging, and central nervous system (CNS) pathologies. The key advantage of the malleability appears to be a functional versatility, owing to which the ECM and PNNs perform diverse functions at specific stages of life. Since functional versatility is predominantly determined by spatiotemporal dynamics, the central question arises; what regulates the ECM and PNN dynamics and thereby critically determines their functions?

Recent studies suggest that the structural organization of ECM and PNNs, and therefore their functions, are regulated by intrinsic mechanisms- driven by neurons- as well as extrinsic- driven primarily by glial cells (Wiese et al., 2012; Rowlands et al., 2018; Crapser et al., 2021; Ribot et al., 2021). CNS glia, including astrocytes, oligodendrocytes, and microglia are capable of producing ECM and PNN components and are significant sources of ECM during development and adulthood (Wiese et al., 2012; Song and Dityatev, 2017). In addition, astrocytes excessively produce ECM molecules under several CNS pathologies, effectuating both protective and detrimental outcomes (Fitch and Silver, 2008; Kim et al., 2016, 2017; George and Geller, 2018). Besides producing ECM molecules of structural and signaling utility, astrocytes release an array of diverse matrix-remodeling proteases and their inhibitors to tightly control the structural integrity of PNNs and ECM (Fitch and Silver, 2008; Patel et al., 2019; Chaunsali et al., 2021).

While astrocytes are mainly engaged with the synthesis and release of ECM and their proteolytic enzymes, it is microglia that contribute significantly to the continuous elimination of the ECM molecules due to their characteristic phagocytic property. Normally, the homeostatic states of ECM and PNNs are maintained by a constitutive expression of ECM and proteases by neurons and astrocytes, as well as clearance by microglia. However, as seen in several recent studies on epilepsy, Alzheimer’s disease (AD), Huntington’s disease (HD), neuropathic pain, etc., dysfunctional microglia leads to abnormal clearance or accumulation of the ECM and PNNs contributing to the pathology (Tewari et al., 2018; Patel et al., 2019; Crapser et al., 2020b,^2021^; Chaunsali et al., 2021; Carceller et al., 2022; Tansley et al., 2022).

In this review, we discuss the classic roles of and recent advances in the functions of ECM and PNNs, followed by the role of glial cells in ECM and PNN remodeling in healthy brain and pathologies. These roles suggest a pivotal contribution of glial cells to this remodeling process and thus encourage a discussion on a glia-centric approach to treatment strategies.

## Structure and functions of extracellular matrix and perineuronal nets in the central nervous system

Extracellular matrix is present in all tissues of the body as a structural framework of amorphous and diffused interstitial matrix; however, brain ECM is unique in its composition and organization. From a composition point of view, a major fraction of the brain ECM consists of glycosaminoglycans (GAGs), proteoglycans, and glycoproteins, with a negligible fraction of fibrous proteins which is contrary to the fibrous protein-rich ECM in a majority of other tissues (McRae and Porter, 2012). Another key feature of the brain ECM is its structural organization into distinct forms such as thin sheets of BMs and highly condensed pericellular coats of PNNs.

### Basement membranes

Basement membrane is an organized ECM assembly in the form of thin sheets that surround the pial surface (meningeal BM) and brain vasculature (vascular BM) (Thomsen et al., 2017). Similar to other forms of ECM, the BMs also show a spatiotemporally dynamic composition which determines their functions at different stages of life. By and large, collagen IV, laminins (1–5), nidogens (1 and 2), and heparin sulfate proteoglycans (HSPGs) (perlecan and agrin) are the most static components (Thomsen et al., 2017). On the other hand, insoluble fibronectin, fibulins, thrombospondins (TSPs), and secreted protein acidic and rich in cysteine (SPARC) are more dynamic and are expressed at specific developmental and pathophysiological states (Thomsen et al., 2017). Besides serving as a major route *via* which fluids and soluble molecules enter and leave the brain, BMs provide structural support by acting as an adhesive substrate for cells to anchor to and mediate signal transduction *via* integrin and other transmembrane matrix receptors (Baeten and Akassoglou, 2011). Meningeal BM is critical for brain development and the absence of the BM or its constituents causes abnormal brain development (Halfter et al., 2002). The vascular BM plays a critical role in maintaining the blood-brain barrier (BBB), as evidenced by BBB disruption and cerebrovascular defects in the absence of BM components such as laminins (Yao et al., 2014) and collagens (Engelhardt, 2003; Jeanne et al., 2015). In several CNS disorders, predominantly in stroke and traumatic brain injury (TBI), BBB disruption is associated with an altered BM, causing an infiltration of otherwise impermeable serum components and immune cells to trigger inflammation and subsequently neuroglial dysfunctions (Thomsen et al., 2017). Extravasation of blood proteins fibrinogen and albumin trigger molecular changes in astrocytes, transforming them into their reactive state which in turn further remodels the ECM and forms glial scars (Kim et al., 2016, 2017) (discussed later).

### Interstitial matrix

Historically, the idea of ECM was pioneered as a neuroglia-embedding structural framework of a diffused, amorphous, and ubiquitously distributed ground substance in the extracellular space (ECS) (Celio, 1999). This form is now known as interstitial matrix and constitutes the highest fraction of brain ECM. Interstitial matrix fills nearly the entire ECS and embeds other phenotypes of ECM such as perineuronal, perisynaptic, and perinodal matrices (Engelhardt, 2003; Lau et al., 2013; Fawcett et al., 2019). The meshwork of the interstitial matrix consists of hyaluronan, proteoglycans, tenascins, link proteins, glycoproteins such as laminins and fibronectin, and a relatively small fraction of fibrous proteins such as collagens and elastin (Rauch, 2007; Lau et al., 2013; Lei et al., 2017). Several transmembrane and membrane-coupled proteins and receptors including CD44, receptor for hyaluronan-mediated motility (RHAMM), Stabilin-2, TNFIP6, SHAP, TLR-2, and TLR-4 are connected directly with the hyaluronan to anchor and stabilize the ECM (Jiang et al., 2011). Similarly, chondroitin sulfate binds to several transmembrane receptors including RPTPσ, LAR, RPTPδ, and Nogo receptors as well as adhesion molecules including NCAM and integrins (Yu et al., 2018). The interstitial matrix harbors ions, secreted molecules such as growth factors and neuromodulatory agents, and most importantly, provides a high hydration capacity to maintain ECS volume and thereby normal brain activity (Perkins et al., 2017; Hrabetova et al., 2018).

A large fraction of diffused interstitial matrix coats the synapses, forming a perisynaptic matrix, and is involved in synaptogenesis and plasticity often under the regulation of matrix remodeling enzymes (Orlando et al., 2012; Korotchenko et al., 2014; Fawcett et al., 2019). Depletion of perisynaptic HA affects synaptic potentiation by altering the lateral mobility of AMPARs (Frischknecht et al., 2009) as well as the activity of L-type voltage-dependent calcium channels (L-VDCCs) at synaptic terminals (Kochlamazashvili et al., 2010). Similarly, Tenascin-C (Tn-C) deficiency impairs synaptic plasticity by altering L-VDCCs signaling, however, Tenascin-R (Tn-R) deficiency, which is expressed around perisomatic synapses, alters NMDAR-dependent LTP by reducing the perisomatic inhibition (Evers et al., 2002; Hayani et al., 2018). More recently, Tn-R appears to be recycled at the active synapse in an activity-dependent manner influencing the synaptic structure (Dankovich et al., 2021). These studies suggest a pivotal role of interstitial matrix molecules in effectuating the dynamic changes at synapses.

Besides PNNs, few other specialized phenotypes of the ECM are embedded largely within the diffused interstitial matrix. For example, perinodal ECM is a condensed form of ECM around the nodes of Ranvier (Bekku and Oohashi, 2019) consisting of Tn-R, brevican, versican, phosphacan, Bral1, and neurocan (Susuki et al., 2013). Tn-R plays an essential role in axonal functions presumably by acting as an ion diffusion barrier (Bekku and Oohashi, 2019) as evidenced by decreased axonal conduction velocity in the optic nerve in Tn-R deficient condition (Weber et al., 1999). Axonal coats are another phenotypic specialization of ECM which are rich in CSPGs, including aggrecan and brevican; however their functional relevance is elusive (Morawski et al., 2012; Jäger et al., 2013). Recent studies support the presence of brevican and NG2 expressing axonal coats surrounding myelinated axons in human brains and are suggested to aid axonal properties (Pantazopoulos et al., 2022).

### Perineuronal nets

**FIGURE 1:**
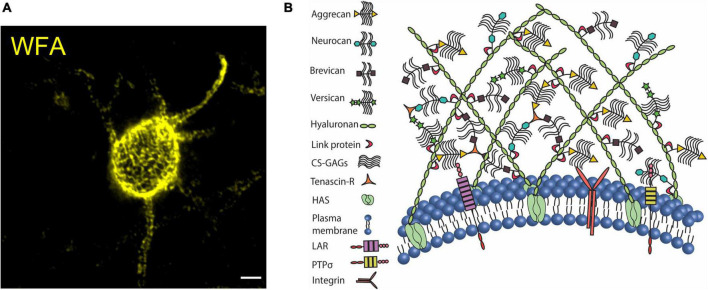
Perineuronal net in mouse cerebral cortex. **(A)** Confocal micrograph of WFA-labeled PNN in the mouse cerebral cortex. PNN coats cell soma, dendrites, and axon initial segment. **(B)** The organization of CSPGs, hyaluronan, link proteins, and tenascin on the plasma membrane forms the assembly of PNN. CSPGs interact with their cell surface receptors to modulate intracellular signaling cascades. Scale bar 5 μm.

Historically, PNNs have been the most intriguing yet enigmatic ECM structures. PNNs are widely expressed in several brain regions including the cerebral cortex, amygdala, striatum, and hippocampus (Morikawa et al., 2017; van’t Spijker and Kwok, 2017; Ulbrich et al., 2021) as well as in the spinal cord (Irvine and Kwok, 2018) of rodents and humans (Chaunsali et al., 2021; Carceller et al., 2022). PNNs are predominantly present on the fast-spiking PV interneurons; however, a small population of other inhibitory and excitatory neurons in brain and spinal cord express PNNs (Irvine and Kwok, 2018; Chaunsali et al., 2021). The typical lattice of PNN is a ternary complex of hyaluronan, link proteins (HAPLNs), proteoglycans of the lectican family or CSPGs including aggrecan, brevican, versican, and neurocan, and tenascin glycoproteins (Tn-C, Tn-R). PNNs can be visualized by fluorescently labeled antibodies that bind to the core proteins or by lectins such as Wisteria floribunda agglutinin (WFA) that bind the GAGs sidechains (Fawcett et al., 2019; Tewari and Sontheimer, 2019; Figure 1A). The cavities of the PNN lattice on the soma, AIS, and dendrites house both excitatory and inhibitory synaptic terminals (Fawcett et al., 2019; Carceller et al., 2020).

The key role of anchoring the extracellular components of PNNs to the cell membrane is performed by HA-producing transmembrane enzymes, hyaluronic acid synthase (HAS 1-3) (Kwok et al., 2011). HAS-associated long chains of HA are connected directly to the link proteins (HAPLN), which in turn bind to the CSPG core proteins. The core proteins of lecticans also form a backbone to which numerous side chains of sulfated GAGs are attached. Lecticans are cross-linked by Tn-R to further secure the assembly (Figure 1B) and a loss of aggrecan crosslinking by Tn-R impairs the PNN assembly around dendrites (Morawski et al., 2014). Despite being a multimolecular assembly, WFA-labeled PNNs remain minimally distorted in the absence of single or multiple PNN components including HA (Arranz et al., 2014), neurocan (Zhou et al., 2001), brevican (Brakebusch et al., 2002), Tn-C (Irintchev et al., 2005; Gottschling et al., 2019), Tn-R (Brückner et al., 2000; Gottschling et al., 2019), and link proteins (Carulli et al., 2010). However, as the one indispensable component of the PNN, aggrecan deficiency leads to the absence of PNNs (Giamanco et al., 2010; Rowlands et al., 2018) (See review Carceller et al., 2022).

In different regions of the developing mouse brain, traces of PNN appear at different ages and gradually achieve fully condensed arborization in several weeks. For example, immature PNNs can be identified in brainstem by postnatal day 4 (Brückner et al., 2000); however in the cerebral cortex and amygdala by postnatal days 14 and 21, respectively (Brückner et al., 2000; Horii-Hayashi et al., 2015). In humans, PNNs appear near 8 weeks in medial prefrontal cortex and mature around 8 years of age (Rogers et al., 2018). By and large, this developmental trajectory of PNN formation coincides with the critical periods of heightened neuroplasticity, in which neuronal circuits are highly responsive to sensory inputs and brain connections are established and strengthened in an activity-dependent manner (Pizzorusso et al., 2002). Sensory deprivation within the critical period permanently disrupts the normal development of the brain circuits; however, sensory deprivation outside the critical period or in adults does not affect neuronal circuits and brain functioning (Hubel and Wiesel, 1965, 1970; Reh et al., 2020). Pioneering studies have shown that preventing PNN formation in the developing brain prolongs the critical period of plasticity, and that disruption of PNNs outside the critical period using a bacterial-derived enzyme Chondroitinase ABC (ChABC) reinstates neuroplasticity similar to that of the critical period, suggesting PNNs as the primary regulator of the critical period plasticity (Pizzorusso et al., 2002; Wang and Fawcett, 2012; Rowlands et al., 2018; Fawcett et al., 2019).

Despite a progressive condensation of CSPGs into PNNs in the developing brain, the total CSPG content remains largely unchanged- which complicates the question of mechanisms whereby PNN CSPGs are inhibitory to neuroplasticity (Miyata et al., 2012). Intriguingly, the plasticity-permissive nature of the developing brain is attributed to a characteristic sulfation pattern of the CSPGs. The developing brain exhibiting immature PNNs and high neuroplasticity possesses a higher C6S proportion than C4S to maintain a low C4S/C6S ratio. Over the developmental period, the ratio changes to a higher C4S/C6S, which not only promotes PNN maturation but also suppresses neuroplasticity (Miyata et al., 2012; Miyata and Kitagawa, 2016; Foscarin et al., 2017). Nevertheless, the downstream cellular and molecular mechanism behind the inhibitory nature of a higher C4S/C6S ratio remains elusive.

Developmental formation of PNNs is activity-dependent, and several brain regions including barrel cortex, thalamus, visual cortex, and vocal center in songbirds show underdeveloped PNNs if deprived of activity, suggesting a high malleability of PNNs (Guimarães et al., 1990; Lander et al., 1997; Pizzorusso et al., 2002; McRae et al., 2007; Nakamura et al., 2009). Although PNNs in the mature CNS appear to be largely stable in a normal physiological state, emerging evidence suggests bidirectional changes in the structure and numerical density of PNNs on a cyclic basis as well as under specific conditions such as drug addiction, maternal hormone fluctuations, and chronic pain (Lasek et al., 2018; Pantazopoulos et al., 2020; Uriarte et al., 2020; Harkness et al., 2021; Mascio et al., 2022).

## Functions of perineuronal nets

Chondroitin sulfate proteoglycans are critical constituents of the PNNs and several signaling functions of CSPGs are independent of their phenotypic appearance as PNNs or interstitial matrix as evidenced in the following studies. In the extensively studied visual system, a high density of CSPGs repels the growing retinal axons to navigate them to their target areas in the developing brain. Conversely, depleting CSPGs with ChABC is disruptive to the axonal guidance and misleads the axons to non-target areas (Brittis et al., 1992; Laabs et al., 2005). Since axonal growth and guidance is a developmental phenomenon, the inhibitory role of CSPGs appears extraneous in adult CNS physiology. However, in CNS injury and trauma, the damaged axons fail to regenerate due to the CSPG-rich glial scar at the injury site and ChABC-mediated removal of CSPGs improves the repair and regeneration and to a certain extent, functional recovery (Silver and Miller, 2004).

Subsequently, in adolescence, CSPGs are condensed as PNNs, which are largely known to lock the synapses to prevent further modifications and close the critical period of heightened neuroplasticity as discussed in the previous section. Intriguingly, the plasticity reinstates in the adult CNS when PNNs are disrupted. Mechanistically, disruption of PNN or its constituents triggers several short and long-term cellular and molecular changes which can promote neuroplasticity. For example, PNN depletion induces synaptic potentiation in otherwise plasticity-resistant CA2 synapses (Carstens et al., 2016). Brevican, a CSPG in the PNNs, is suggested to regulate the localization of potassium channels and α-amino-3-hydroxy-5-methyl-4-isoxazolepropionic acid (AMPA) receptors expression on PV cells (Favuzzi et al., 2017). At the network level, PNN depletion affects gamma oscillations (30–80 Hz) (Lensjø et al., 2017b) and sharp wave ripples (SWRs) (Sun et al., 2018). Since PV neuron activity is pivotal for the generation of gamma oscillations and SWRs, it is plausible that neuroplasticity upon PNN depletion is partly effectuated by the altered activity of PV neurons. These functional changes due to PNN disruption are also accompanied by structural changes at synapses, including alterations in the numbers of synaptic contacts, spine dynamics, and expression of ion channels and receptors (Frischknecht et al., 2009; Favuzzi et al., 2017; Carceller et al., 2020). These studies suggest a variety of ways by which PNN disruption can effectuate the synaptic plasticity.

Perineuronal nets and interstitial matrix are by and large composed of the same set of ECM molecules; therefore several functions of PNNs can be considered independent of their structural integrity. However, there are several functions which require the organized PNN assembly with sulfated proteoglycans (Miyata and Kitagawa, 2016; Fawcett et al., 2019). Several signaling proteins including OTX2, Semaphorin 3a, Narp, and reelin are trapped in the PNN lattice and activate intracellular signaling cascades to facilitate the developmental maturation of PV neurons (Fawcett et al., 2019). The condensed PNN has a high density of negative charge which protects PV neurons from extracellular stressors (Suttkus et al., 2014), which is markedly evidenced in schizophrenia (Cabungcal et al., 2013, 2014) and AD wherein PV neurons are relatively spared due to their PNN coats (Morawski et al., 2010, 2012).

The sulfated proteoglycans on the PNNs constitute a high-density cloud of negative charges around the PV cells which can attract a high concentration of Na^+^, K^+^, or Ca^++^ ions. During the fast-spiking activity of PV neurons, a dynamic exchange of Na^+^ and K^+^ ions with the stationary negative charges of the PNNs can aid the PV neuron activity (Härtig et al., 1999; Morawski et al., 2015). Besides the ion buffering in the ECS, PNNs can also directly influence the spiking properties of the PV neurons as shown by us (Tewari et al., 2018) and others (Balmer, 2016; Wingert and Sorg, 2021). The pioneering *in vitro* (Dityatev et al., 2007) and more recent studies on hippocampal fast-spiking interneurons *in situ* brain slices (Favuzzi et al., 2017; Hayani et al., 2018) report a lower firing threshold without any changes in passive neuronal properties, however, few other studies show a reduced spiking upon PNN disruption (Balmer, 2016; Tewari et al., 2018). This ambiguity can be attributed to several factors including PNN disruption methods, brain regions, experimental design, resting state, and excitatory/fast-spiking type of PNN-expressing neurons, as discussed in detail by Wingert and Sorg (2021).

In a mouse model of human glioma-associated epilepsy, we observed that disruption of cortical PNNs by glioma-released matrix metalloproteinases (MMPs) increases the membrane capacitance of the PV neurons leading to a reduction in spike firing activity and consequently reducing the overall inhibitory drive. Experimental disruption of PNNs mimics the increased capacitance and reduced firing activity of PV neurons as shown by PV neurons with disrupted PNNs in glioma (Tewari et al., 2018), suggesting a pivotal role of PNNs in aiding the fast-spiking properties of PV neurons. PNNs seem to determine the activity of excitatory neurons equally well, as evidenced in a recent study in which microglia-mediated degradation of PNNs around excitatory projection neurons in the spinal cord enhances their activity and induces pain-related behavior (Tansley et al., 2022). Another example is the induction of synaptic plasticity in CA2 neurons upon their PNN depletion, which are otherwise resistant to potentiation (Carstens et al., 2016).

The necessity of PNNs in CNS functioning is profoundly evidenced in CNS disorders in which PNN disruption is commonly observed; experimental PNN disruption largely phenocopies the disease characteristics. In acquired forms of epilepsies triggered by injury, stroke, and brain tumors, elevated matrix remodeling proteases disrupt the PNNs (McRae et al., 2012; Rankin-Gee et al., 2015; Kim et al., 2016; Dubey et al., 2017; Tewari et al., 2018; Patel et al., 2019). PNN disruption potentially exposes the PV neurons to heightened oxidative stress (Cabungcal et al., 2013), leading to a reduction in the overall abundance of PV neurons and thereby further lowering the inhibitory drive as shown by us (Tewari et al., 2018) and others (Enwright et al., 2016; Hatcher et al., 2020). Elimination of PNN and its constituents not only increases the propensity of seizure and epileptiform activity in excitatory neurons, but also causes spontaneous seizures (Arranz et al., 2014; Rempe et al., 2018; Tewari et al., 2018; Patel et al., 2019). These studies support the idea that PNN disruption is not only able to generate neuronal hyperexcitability, but that PNN disruption due to CNS insults can contribute to the process of epileptogenesis by PV neuron dysfunction and altered inhibition.

A similar dysfunction of PV neurons accompanied by disrupted PNNs is evidenced in animal models and human subjects of schizophrenia, bipolar disorder, and autism spectrum disorders (Pantazopoulos and Berretta, 2016; Fawcett et al., 2019), wherein preventing PNN disruption by blocking MMP activity largely ameliorates disease symptoms (Levkovitz et al., 2009; Khodaie-Ardakani et al., 2014). Several recent studies on CNS disorders including AD (Abbott and Kepler, 1990; Fawcett et al., 2019; Crapser et al., 2020b), HD (Crapser et al., 2020a,^2021^), multiple sclerosis (MS) (Lau et al., 2013), and schizophrenia (Pantazopoulos et al., 2010; Mauney et al., 2013) also show altered PNN in key brain areas. Notably, glial contribution in PNN remodeling is explicitly evidenced in many of these studies as discussed in later sections.

In summary, a large number of studies suggest that the physiological functions of the PNNs and their constituents broadly encompass developmental signaling, regulation of neuroplasticity, modulation of neuronal activity, extracellular ion homeostasis, and neuroprotection. In diseased states increased proteolytic cleavage of PNNs disrupts their structural integrity and reduces the overall abundance. Depending on the brain area/s involved, loss of PNNs can contribute to disease etiology predominantly by PV neuron dysfunction and altered E-I balance, loss of neuroprotection, altered ECS and ionic balance, and maladaptive neuroplasticity (Reichelt et al., 2019). The causal role of PNNs in E-I imbalance and ECM and ionic homeostasis in epilepsy appears to be convincing; meanwhile, the causal role of PNNs in many neuropsychiatric and neurodegenerative disorders is still in its infancy. A vast majority of the studies on the PNNs use ChABC or hyaluronidase enzymes which indiscriminately cleave the GAGs of PNNs and interstitial matrix. This lack of tools to selectively manipulate PNNs is a major limitation in the field.

## Homeostatic regulation of extracellular matrix and perineuronal net by central nervous system glia

One of the classic housekeeping functions of glial cells is the continuous secretion of ECM molecules to maintain the architecture and extracellular milieu of the CNS. In principle, both neurons and glial cells synthesize and secrete ECM molecules; however, glial cells- especially astrocytes and microglia- are the primary regulators of ECM wear and tear in CNS pathophysiology. In this section, we discuss the role of glial cells in the homeostatic regulation of ECM and PNN and consequently the functional outcome.

**FIGURE 2:**
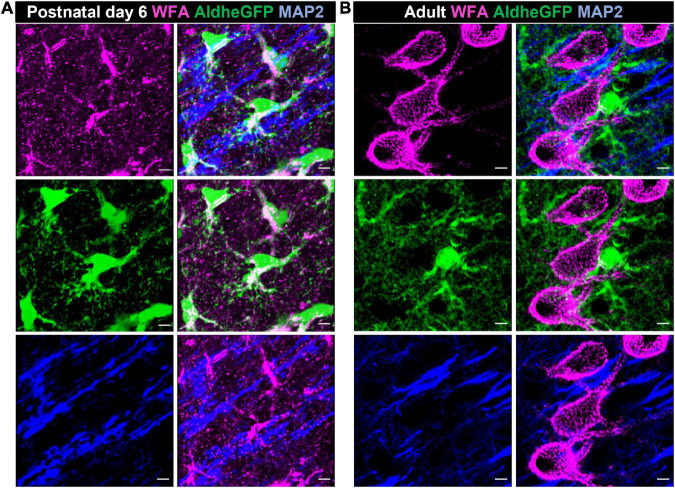
Astrocytes produce CSPGs in the developing brain. Confocal micrographs showing WFA reactivity (magenta – top left), GFP-immunolabelled aldheGFP-expressing astrocytes (green – middle left) and neuronal marker MAP2 (blue- bottom left) in postnatal day 6 **(A)**, and adult **(B)** mouse cerebral cortex. In postnatal day 6 **(A)**, WFA reactivity predominantly colocalizes with GFP-labeled astrocytes (**A**, middle right) compared to the MAP-2 labeled neuronal processes (**A**, bottom right). In adult, WFA reactivity is present only in PNNs (**B**, top left); astrocytic (**B**, middle right) and neuronal (**B**, bottom right) processes show no detectable WFA reactivity. Scale bar 5 μm.

**FIGURE 3:**
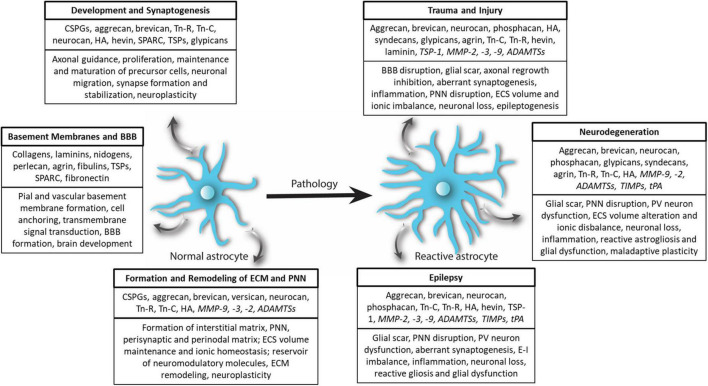
Extracellular matrix remodeling by astrocytes in physiology and pathology. In physiological conditions, normal astrocytes release several ECM molecules to govern diverse processes associated with CNS development, synaptogenesis, basement membrane and BBB formation as well as formation of the structural scaffold of brain ECM and PNN. Astrocytes also release several proteases and regulatory molecules (italicized) to remodel ECM and PNNs to maintain homeostatic neuroplasticity. In CNS pathologies, most prominently in trauma and injury, epilepsy, and neurodegenerative diseases, normal astrocytes turn reactive and remodel ECM and PNN by accumulating ECM or degrading ECM and PNN by releasing proteases (italicized) thereby effectuating beneficial and deleterious outcomes.

Astrocytes, the most abundant CNS glia, are involved in a variety of functions including neuronal migration, secretion of growth factors and neuromodulatory molecules, synaptogenesis, synaptic pruning, and water, ion, and neurotransmitter homeostasis (Phatnani and Maniatis, 2015; Patel et al., 2019; Alcoreza et al., 2021). In the developing brain, astrocytes are the predominant source of ECM molecules including CSPGs, HA, and tenascins, which in turn serve both structural and signaling roles (Figures 2, 3). By varying the spatiotemporal expression of CSPGs and Tn-C, astrocytes regulate the proliferation, maintenance, and maturation of neuronal precursor stem cells and oligodendrocyte precursor cells (OPCs) as well as neuronal migration, neurite outgrowth, extension, and guidance (Powell et al., 1997; Powell and Geller, 1999; Wiese et al., 2012; Amin and Borrell, 2020; Somaiya et al., 2022). Tn-C appears to be expressed by radial glia as well as differentiated astrocytes, and additionally regulates the proliferation of astrocyte progenitor cells (Karus et al., 2011). In a quadruple knockout mouse, astrocyte-derived Tn-C, Tn-R, brevican, and neurocan have been reported to control synapse formation and stabilization (Geissler et al., 2013; Figure 3).

Astrocytes are the predominant source of hyaluronan that constitutes the interstitial matrix and PNNs in the gray matter and surrounds the myelinated fibers in white matter (Cargill et al., 2012; Peters and Sherman, 2020). Since hyaluronan chains of the PNN extend from the neuronal surface-bound HAS (Fawcett et al., 2019), neurons appear to be a predominant source of PNN-forming hyaluronan. Generally, hyaluronan is secreted as a high molecular weight (HMW) polymer which interacts with HA receptors such as CD44, RHAMM, or LTR2; and triggers proliferation, differentiation, and migration of the stem cell in developing brain (Lindwall et al., 2013; Peters and Sherman, 2020). In the adult brain, astrocytes produce hyaluronan as well as hyaluronan-cleaving enzyme hyaluronidase in the subventricular zone, where adult stem cells reside. Thus, by regulating the HA catabolism, astrocytes are speculated to keep stem cells in a quiescent state (Lindwall et al., 2013). In pathologies such as trauma and injury, HMW hyaluronan can be cleaved into short fragments of low molecular weight by hyaluronidases and MMPs (Peters and Sherman, 2020), which by and large have distinct and sometimes opposite biological effects. By expressing both hyaluronidase and MMPs, astrocytes play an important role in hyaluronan synthesis and catabolism (Muir et al., 2002; Al’Qteishat et al., 2006). HA in turn regulates the morphology of astrocytes and proper trafficking and function of glutamate transporters by CD44-evoked Rac1 signaling (Hayashi et al., 2019; Peters and Sherman, 2020).

Besides CSPGs, tenascins, and HA, which act as both structural and signaling molecules, astrocytes secrete several ECM glycoproteins known as matricellular proteins primarily for a signaling function. By adjusting the spatiotemporal expression of matricellular proteins such as hevin/SPARC (Kucukdereli et al., 2011), thrombospondin (TSP) (Christopherson et al., 2005), and Glypican 4 and 6 (Allen et al., 2012), astrocytes control excitatory synaptogenesis in the developing CNS (Figure 3). The expression of the majority of matricellular proteins decreases during the later phase of postnatal development; however reactive astrocytes upregulate their expression in several pathological conditions, potentially causing aberrant synaptogenesis and maladaptive plasticity (Jones and Bouvier, 2014; Kim et al., 2016).

From a structural point of view, astrocytes, in conjunction with capillary endothelial cells and pericytes, synthesize and assemble ECM components to form the BM (Yao et al., 2014; Thomsen et al., 2017). Subsequently, astrocytic perivascular endfeet- in association with the BM and pericytes- form the structural basis of the BBB, for whose maintenance astrocytic laminin is indispensable (Thomsen et al., 2017). In the parenchymal space, astrocytes secrete hyaluronan, CSPGs- including brevican, neurocan, versican, and aggrecan- and Tn-C in order to form and maintain interstitial matrix (Wiese et al., 2012; Patel et al., 2019).

Although astrocytes and neurons produce aggrecan, which in turn orchestrates PNNs, the contribution of astrocytic aggrecan in PNN formation and maintenance can be questioned (Song and Dityatev, 2017) as evidenced by the absence of PNNs in neuron-specific aggrecan knockout (Rowlands et al., 2018) and formation of ECM coatings *in vitro* without astrocytes (Miyata et al., 2005; Giamanco and Matthews, 2012). However, how critical astrocytes are in maintaining the PNNs *in vivo* is a frontier area of investigation. A recent study suggests that astrocytes are key regulators of PNN maturation and thereby critical period plasticity. During the critical period, immature astrocytes increase connexin 30 levels, which subsequently activates the RhoA-ROCK pathway to suppress MMP-9 expression and allows PNN condensation and PV cell maturation (Ribot et al., 2021). These studies suggest that although astrocytes do not directly synthesize PNNs, they indirectly regulate their developmental formation.

Other CNS glial cells such as OPCs and oligodendrocytes, and microglia are also a source of ECM, but in a more restricted manner (Pu et al., 2018). For example, oligodendrocytes and OPCs deposit a peculiar ECM rich in Tn-C and Tn-R around the node of Ranvier termed the perinodal ECM, which is essential for the clustering of Na^+^ channels (Susuki et al., 2013; Fawcett et al., 2019). OPCs secrete brevican during myelination and are suggested to form axonal coats around myelinated axons (Pantazopoulos et al., 2022). Besides being a source of CSPGs, oligodendrocyte lineage cells are critically susceptible to CSPGs as evidenced by inhibition of OPCs migration and maturation into oligodendrocytes especially near the demyelinated lesion (Lau et al., 2013). Microglia can, under special circumstances, produce ECM molecules including CSPGs; however, their contribution to the formation of brain ECM and PNNs is not entirely clear (Lau et al., 2013; Pu et al., 2018). These studies suggest that glial cells are major sources of ECM molecules whereby ECM constituents are used as building blocks of brain architecture as well as signaling molecules to regulate brain development. How glial cells remodel ECM in CNS pathologies and the consequences of said remodeling are discussed in the next sections.

## Extracellular matrix and perineuronal net remodeling by astrocytes in central nervous system pathologies

In nearly all CNS pathologies, astrocytes respond by undergoing morphological, molecular, and functional changes that turn them into reactive astrocytes (Escartin et al., 2021). Upregulation of glial fibrillary acidic protein (GFAP), an intermediate filament protein, is the most common molecular change and has been used widely as a marker for reactive astrocytes (Patel et al., 2019; Escartin et al., 2021). In a broader sense, reactive astrocytes influence ECM and PNN homeostasis by altering the expression levels of ECM as well as the matrix remodeling proteases including MMPs. As a consequence, in several CNS pathologies such as acquired epilepsies, TBI, MS, and glioma, the interstitial matrix is upregulated; however, the PNNs are disrupted or lost (Lau et al., 2013; George and Geller, 2018; Rempe et al., 2018). This contrasting fate can be attributed to the glial upregulation of ECM molecules at the injury and increased expression of matrix-degrading proteases in the surrounded areas. Interestingly, CNS disorders without a focal injury such as schizophrenia also show glial ECM abnormalities in conjunction with PNN disruption in several key brain areas suggesting a potential role of glia in PNN reduction (Pantazopoulos et al., 2010, 2015; Mauney et al., 2013). Considering its widespread prevalence, ECM remodeling appears to be a generic response of reactive astrocytes to a majority of brain disorders (Figure 3).

The deleterious consequences of ECM remodeling by glial cells are remarkable in CNS pathologies with a focal lesion such as ischemia, glioma, MS lesion, and brain and spinal cord injury. At the focal lesion or injury site, astrocytes turn reactive (Escartin et al., 2021) and form a barrier or glial scar, aided by infiltration of microglia, macrophages, meningeal cells, and fibroblast (Rhodes et al., 2003). Scar-forming reactive astrocytes accumulate a variety of ECM molecules, including CSPGs, HA, tenascins, fibronectins, and laminins, and isolate the insult from surrounding areas to limit the spread of inflammation and tissue damage (Kim et al., 2016). In the case of traumatic injuries, especially in the spinal cord, reactive astrocytes deposit various CSPGs in the glial scar which inhibits neuronal recovery and axonal regrowth (Silver and Miller, 2004; Lau et al., 2013). Similarly, in cortical injury, reactive astrocytes highly upregulate CSPGs and inhibit cortical axonal regeneration (McKeon et al., 1999; Busch and Silver, 2007; Kim et al., 2016). ChABC application to dissolve CSPGs enhances axonal growth and functional recovery to great extent (Bradbury et al., 2002; Huang et al., 2006; Lee et al., 2010). Interestingly, astrocyte specific ChABC expression in transgenic mice reduced CSPG expression after spinal cord injury and enhanced axonal growth and recovery (Cafferty et al., 2007). These studies provide compelling evidence of inhibitory functions of glial-derived CSPGs and can be harnessed to generate glia-centric therapeutic tools.

Reactive astrocytes also release non-sulfated proteoglycan HA in the glial scar areas in the brain or spinal cord lesions, evoking beneficial and deleterious effects (Sherman et al., 2015). The HMW HA accumulation after spinal cord injury suppresses the activation of astrocytes as well as glial scar formation. On the other hand, low MW hyaluronan accumulation [perhaps as a cleavage product of HMW HA due to increased hyaluronidase activity (Al’Qteishat et al., 2006)] promotes astrocytic activation and proliferation (Struve et al., 2005). Following an ischemic stroke, scar-forming astrocytes upregulate the hyaluronan as well as HA receptor RHAMM, and are speculated to evoke the migration of adult stem cells from the subventricular niche to the ischemic site for repair (Lindwall et al., 2013). Glial upregulation of HA is also evidenced in aging; however, the downstream effects require thorough investigation (Peters and Sherman, 2020).

Similarly to CNS trauma and injury, in MS several ECM components such as hyaluronan, CSPGs (aggrecan, versican, and neurocan), and glycoprotein (fibronectin and vitronectin) are deposited around the lesion, primarily by reactive astrocytes forming the glial scar (Lau et al., 2013). Condensed CSPGs and HA at the lesion prevent OPC growth, differentiation, and migration to the lesion site, thereby making remyelination nearly impossible. Despite providing beneficial effects to the axon growth in spinal cord injury, HA and CSPG disruption strategies using exogenous MMPs, ChABC or hyaluronidase have not proven promising, largely due to indiscriminate disruption of both permissive and non-permissive ECM components and generation of non-permissive cleavage product (Lau et al., 2013). Since reactive astrocytes are the major CSPG and HA-producing cells at the lesion, strategies to manipulate the cellular machinery of CSPG and HA biosynthesis and release can be speculatively proposed as an alternative approach.

Since PNN disruption is commonly observed in several CNS disorders, it is a legitimate question whether astrocytes play any role in PNN disruption in such pathologies. The answer lies partly in the fact that astrocytes are a major source of PNN-disrupting MMPs in both homeostatic and diseased states. After brain injury and trauma, PNN disruption is accompanied by upregulation of MMP-2 and MMP-3 levels by reactive astrocytes (Muir et al., 2002; Falo et al., 2006; Lorenzo Bozzelli et al., 2018). MMP-9 appears to be the major effector of PNN disruption triggered by activation of TGFβ signaling in reactive astrocytes after albumin extravasation due to BBB disruption in various neurological insults (Kim et al., 2017). Notably, microglia, infiltrating immune cells, and neurons are also sources of MMPs after CNS insults; therefore astrocytes may not be solely responsible for PNN disruption (Rosenberg, 2002). This fact is also supported by our study on glioma-associated epilepsy, in which PNN disruption did not spatially correlate with reactive astrogliosis and astrocytes did not show MMP activity (Tewari et al., 2018).

## Extracellular matrix and perineuronal net remodeling by microglia in central nervous system pathologies

Microglia are the resident immune cells in the brain and are known for their classic phagocytic function of eliminating entire cells or subcellular structures- mainly synapses- in healthy and diseased states (Wolf et al., 2017). The developing brain forms an excessive number of synapses, which are subsequently eliminated by a process known as synaptic pruning, in which microglia play a pivotal role. In a healthy adult brain, microglia also actively prune synapses in an effort to aid neuronal plasticity (Wolf et al., 2017). Besides these well-known functions, recent studies suggest a conversion of normal astrocytes to a detrimental neurotoxic phenotype under the influence of microglia-released cytokines IL-1α, TNF-α, and complement protein C1q in pathological conditions (Liddelow et al., 2017).

Pioneering studies in the recent past implicate microglia equally well in the regulation of homeostatic functions such as ECM and PNN remodeling, eventually governing synaptic plasticity. The classic example is a reversible increase in the abundance and condensation of cortical PNNs (Crapser et al., 2020a; Liu et al., 2021) and increased brevican accumulation around hippocampal synapses upon elimination of microglia in normal CNS (Strackeljan et al., 2021). PNN and ECM disruption is evidenced in CNS pathologies such as AD, wherein microglia appear to effectuate the clearance of the ECM and PNN components either directly, by stripping PNNs from the neuronal surface; or indirectly, by clearing the PNN debris after proteolytic cleavage by MMPs (Fawcett et al., 2019; Crapser et al., 2020b,^2021^). Interestingly, eliminating microglia effectively restores the ECM components as well as PNN (Crapser et al., 2020a,b) and can be explored further from a therapeutic angle.

A similar role of microglia has recently been reported in the spinal cord dorsal horn wherein peripheral nerve injury promotes microglial degradation and endocytosis of PNNs around projection neurons. PNN depletion activates the projection neurons to induce pain-related behavior (Tansley et al., 2022). Despite such explicit presentations of microglial involvement, what triggers microglia to start stripping PNNs and ECM components with or without proteolytic cleavage is still an open question. At a minimum, it can be speculated that homeostatic PNN regulation can also be regulated by neuronally released cytokine interleukin-33 (IL-33) which guides microglia to clear ECM to facilitate homeostatic synaptic plasticity; a loss of IL-33 signal leads to ECM accumulation around synapses and dendrites (Nguyen et al., 2020). Similarly, in the developing brain, astrocytes are the main source of IL-33, which in turn instructs microglia to engulf redundant synapses (Vainchtein et al., 2018). Another study revealed the role of microglial protease cathepsin-S in maintaining a diurnal rhythm of the PNN labeling by demonstrating modification in PNNs in a circadian manner that coincides with the rhythmic expression of protease cathepsin-S (Pantazopoulos et al., 2020); however, cyclic rhythm of PNNs is still debated (Barahona et al., 2022). Collectively these and several other recent studies (Venturino et al., 2021) suggest microglia as an integral element of the ECM and PNN remodeling in homeostasis and diseased states; however, the mechanistic insight and therapeutic potential warrant further investigation.

## Metalloproteinases: Main effectors of extracellular matrix and perineuronal net remodeling

Despite a wide range of upstream cellular and molecular events, the mechanisms of ECM and PNN remodeling largely converge onto the metalloproteinases, which serve as an immediate effector to decisively control the ECM and PNN remodeling. Metalloproteinases are zinc-containing endopeptidases, normally expressed by neurons, astrocytes, microglia, and endothelial cells. However, under pathological conditions such as ischemic stroke and brain tumors, non-resident immune cells and tumor cells can also produce metalloproteinases (Tewari et al., 2018; Patel et al., 2019; Chaunsali et al., 2021). Proteolytic remodeling of ECM by metalloproteinase is pivotal in regulating multiple physiological and pathophysiological processes during CNS development and survival, angiogenesis, neurogenesis, axonal growth and regeneration, CNS injury, tumor metastasis, and neuroinflammation (Gottschall and Howell, 2015; Rempe et al., 2016).

Metalloproteinases consist of two families: MMP and a disintegrin and metalloproteinase with thrombospondin motifs (ADAMTSs). In principle, out of 24 MMPs, MMP-1–3, 7–17, 24, and 28 are expressed in the brain under specific pathophysiological circumstances; however, a vast majority of them remain largely undetectable in a normal physiological state (Rempe et al., 2016; Beroun et al., 2019). Based on substrate specificity, major MMPs can be categorized into collagenase (MMP-1, 8, 13); gelatinase (MMP-2, 9) which can degrade collagen IV, fibronectin, laminin, aggrecan, gelatin, elastin, and non-matrix substrates; and stromelysins (MMP-3, 10, 11) which can cleave collagens I, III, IV, V, IX, and X (Nakamura et al., 2009), fibronectin, denatured collagens, laminin, and cartilage proteoglycans (Chang et al., 2016). ADAMTSs are equally diverse with 19 family members, of which ADAMTSs 1, 4, 5, 9, and 15 are expressed in specific brain regions under different circumstances (Gottschall and Howell, 2015). ADAMTSs1, 4, and 5 are known as proteoglycanases or aggrecanases as they can cleave major brain proteoglycans aggrecan, brevican and versican (Kelwick et al., 2015). By and large, ADAMTSs complement the homeostatic and pathological functions of MMPs in developmental ECM remodeling, tissue repair after injury, inflammation, and cancers (Song and Dityatev, 2017; Testa et al., 2019).

Matrix metalloproteinases and ADAMTSs can collectively cleave all elements of the ECM and PNNs as well as several growth factors and membrane proteins (See review Yong et al., 2001; Lu et al., 2011; Gottschall and Howell, 2015); thus, their upstream and downstream regulations are critically important. Indeed, the activity of metalloproteinase is tightly regulated at multiple levels, which makes them ideal candidates for spatiotemporally controlling the ECM and PNN remodeling. At the transcriptional level, most of the MMPs are expressed non-constitutively and need some form of extrinsic signals to trigger their transcription, such as inflammatory cytokines, growth factors, chemokines, and cell-cell or cell-matrix interactions. Several MMPs are expressed as inactive zymogens and need an activation process; this is predominantly cleavage of a propeptide region by intra- and extracellular proteases, including other MMPs. These post-translation modifications bring another level of MMP activity regulation. Even after MMPs are synthesized, secreted, and activated, tissue inhibitors of metalloproteinases (TIMP1-4) can inactivate them by reversibly binding to their catalytic site and thereby terminating the proteolytic activity (Yong et al., 2001). Among other extrinsic regulators, activation of neurotransmitter receptors for glutamate (Dziembowska et al., 2012), dopamine (Mitlöhner et al., 2020), and serotonin (Bijata et al., 2017) are shown to directly regulate MMP-9 and ADAMTS-4 and -5 activity and subsequently trigger neuroplasticity.

From the brain ECM perspective, MMP-2 and MMP-9, and to a lesser extent MMP-3, are the most studied metalloproteinases, perhaps due to their reliable detection and measurement methods and also their involvement in multiple processes (Dzwonek et al., 2004). MMP-2, or gelatinase A, is expressed by both neurons and glial cells; however, astrocytes appear to be the major source (Dzwonek et al., 2004; Brkic et al., 2015). Functionally, mostly MMP-2, along with MMP-9, is involved in several processes including neurogenesis, migration, dendritic and axonal outgrowth, and guidance in the developing brain as well as migration, regeneration, adult neurogenesis, and angiogenesis in the adult brain (see review Verslegers et al., 2013). In several CNS pathologies in which PNN disruption and ECM remodeling is commonly observed- including ischemia, traumatic brain injury, and seizures- MMP-2 levels, especially in glial cells, are also upregulated (Dzwonek et al., 2004; Kim and Hwang, 2011; Verslegers et al., 2013; Quattromani et al., 2018). Despite such explicit correlation, the decisive contribution of astrocytic MMP-2 in PNN disruption is hard to discern due to a concomitant release of MMP-9 from neurons which can also degrade PNNs (Kim and Hwang, 2011).

MMP-9 is another gelatinase expressed by neurons, astrocytes, and oligodendrocytes (Kamat et al., 2014; Song and Dityatev, 2017) and is the most widely implicated metalloproteinase in CNS homeostasis and pathologies. MMP-9 appears to be the key player in ECM and PNN remodeling-induced neuroplasticity in physiological and pathological conditions. For example, MMP-9 and MMP-2 loosen PNN after enriched environment rearing (Foscarin et al., 2011) or light exposure after monocular deprivation (Wen et al., 2018b) to facilitate neuroplasticity. MMP-9 plays a pivotal role in hippocampal-dependent plasticity, possibly by ECM degradation and inducing surface recruitment of NMDAR between non-synaptic and synaptic compartments, activation of L-VDCCs, and dendritic spine modifications (Kochlamazashvili et al., 2010; Verslegers et al., 2013). A recently reported fundamental role of astrocytes in closing the critical period of visual plasticity is effectuated by MMP-9. Astrocytic connexin 30 signaling suppresses MMP-9 expression to allow maturation of PNNs, thereby closing the critical period of visual plasticity (Ribot et al., 2021). On the other hand, reactive astrocytes in CNS pathologies upregulate MMP-9 *via* inflammatory signaling (Kim et al., 2016). MMP-9 upregulation correlates remarkably with disruption of the PNN in an overwhelming number of studies on epilepsy (McRae and Porter, 2012; Kim et al., 2016, 2017; Tewari et al., 2018; Chaunsali et al., 2021), TBI and stroke (Kim et al., 2017), glioblastoma (Tewari et al., 2018; Hatcher et al., 2020), neurodegenerative (Kamat et al., 2014; Brkic et al., 2015) and neuropsychiatric diseases, and blocking MMP-9 activity generally prevents PNN disruption and improves the disease pathologies (Testa et al., 2019). More recently, inhibition of MMP-2/9 by IPR-179 showed promising antiseizure and antiepileptogenic effects as well as improved cognitive deficits induced by seizures (Broekaart et al., 2021). Similarly, in genetic disorders including Fragile X Syndrome, elevated MMP-9 correlates with PNN disruption, and genetic reduction of MMP-9 promotes PNN formation (Wen et al., 2018a). Overall, MMP-2 and MMP-9 are primary effectors of neuronal and glial cell mediated ECM and PNN remodeling in health and diseases.

MMP-3 or stromelysin-1 is an emerging ECM regulator due to its broad substrate specificity, particularly in its ability to activate pro-MMPs including MMP-2 and MMP-9 (Kim and Hwang, 2011). Perhaps due to this upstream control ability, MMP-3 level in healthy neurons is very low to undetectable. In pathological conditions, neurons, oligodendrocytes, astrocytes, and microglia release MMP-3, which is implicated in neuroinflammation and apoptosis associated with several neurodegenerative disorders (Rempe et al., 2016). Compared to MMP-9, the roles of MMP-3 in context to PNN remodeling and neuroplasticity in adult brains are not explicitly studied. However, MMP-3 has largely been associated with detrimental processes including inflammation, apoptosis, BBB breakdown, neurodegeneration, and demyelination (Kim and Hwang, 2011; Van Hove et al., 2012).

Besides metalloproteinases, CSPGs and PNNs can also be cleaved by tissue-type plasminogen activator (tPA) which is a serine protease and classically known to dissolve blood clots. tPA can degrade PNN directly as its substrate or *via* activating MMPs and upregulated tPA levels may be associated with disrupted PNNs in ischemia and epilepsy (McRae and Porter, 2012; Quattromani et al., 2018). However, whether the homeostatic tPA expression can remodel PNNs is not known. Endogenous hyaluronidase is another enzyme that can degrade PNNs indirectly by cleaving the hyaluronan backbone of the PNNs. Although hyaluronidase levels are elevated after stroke and brain injury, not much is known about their role in the homeostatic and pathological remodeling of ECM and PNNs (Sherman et al., 2015; Reed et al., 2019).

## Concluding remarks

Extracellular matrix forms the structural scaffold of the brain architecture and serves both structural and signaling functions in the developing and adult brain. PNNs are a highly organized form of ECM and confer several functions including neuroplasticity, ionic homeostasis, neuroprotection, and regulation of neuronal activity. A growing body of evidence causally links PNN disruption with the pathophysiology of several CNS disorders such as epilepsy. Glial cells, predominantly astrocytes, are one of the major sources of ECM molecules in developing and adult CNS, thereby directly influencing brain development and functions. Glial cells also release matrix cleaving proteases which are the main effectors of ECM and PNN remodeling during development, adulthood, aging, and diseases. By regulating the spatiotemporal expression of ECM molecules as well as the ECM remodeling proteases, glial cells play a central role in ECM homeostasis in CNS physiology and pathology. Owing to their multifaceted roles in ECM homeostasis, glial cells appear to be promising targets for therapeutic interventions. However, more studies are required to understand the mechanistic insight of glial regulation of ECM and PNNs to pinpoint the molecular pathways and target molecules.

## Author contributions

BT: literature search, data curation, and manuscript writing, editing, and communication. LC and CP: literature search, data curation, and manuscript editing. HS: literature search, manuscript editing, and funding acquisition. All authors contributed to the article and approved the submitted version.

## References

[B1] Abbott L. Kepler T. B. (1990). *Model Neurons: From Hodgkin-Huxley to Hopfield. Statistical Mechanics of Neural Networks.* Berlin: Springer, 5–18. 10.1007/3540532676_37

[B2] Alcoreza O. B. Patel D. C. Tewari B. P. Sontheimer H. (2021). Dysregulation of ambient glutamate and glutamate receptors in epilepsy: An astrocytic perspective. *Front. Neurol.* 12:652159. 10.3389/fneur.2021.652159 33828523 PMC8019783

[B3] Allen N. J. Bennett M. L. Foo L. C. Wang G. X. Chakraborty C. Smith S. J. (2012). Astrocyte glypicans 4 and 6 promote formation of excitatory synapses via GluA1 AMPA receptors. *Nature* 486 410–414. 10.1038/nature11059 22722203 PMC3383085

[B4] Al’Qteishat A. Gaffney J. Krupinski J. Rubio F. West D. Kumar S. (2006). Changes in hyaluronan production and metabolism following ischaemic stroke in man. *Brain* 129 2158–2176. 10.1093/brain/awl139 16731541

[B5] Amin S. Borrell V. (2020). The extracellular matrix in the evolution of cortical development and folding. *Front. Cell Dev. Biol.* 8:604448. 10.3389/fcell.2020.604448 33344456 PMC7744631

[B6] Arranz A. M. Perkins K. L. Irie F. Lewis D. P. Hrabe J. Xiao F. (2014). Hyaluronan deficiency due to Has3 knock-out causes altered neuronal activity and seizures via reduction in brain extracellular space. *J. Neurosci.* 34 6164–6176. 10.1523/JNEUROSCI.3458-13.2014 24790187 PMC4004806

[B7] Baeten K. M. Akassoglou K. (2011). Extracellular matrix and matrix receptors in blood-brain barrier formation and stroke. *Dev. Neurobiol.* 71 1018–1039. 10.1002/dneu.20954 21780303 PMC3482610

[B8] Balmer T. S. (2016). Perineuronal nets enhance the excitability of fast-spiking neurons. *eNeuro* 3 112–116. 10.1523/ENEURO.0112-16.2016 27570824 PMC4987413

[B9] Barahona R. A. Morabito S. Swarup V. Green K. N. (2022). Cortical diurnal rhythms remain intact with microglial depletion. *Sci. Rep.* 12:114. 10.1038/s41598-021-04079-w 34997092 PMC8742049

[B10] Bekku Y. Oohashi T. (2019). Under the ECM Dome: The physiological role of the perinodal extracellular matrix as an ion diffusion barrier. *Adv. Exp. Med. Biol.* 1190 107–122. 10.1007/978-981-32-9636-7_8 31760641

[B11] Beroun A. Mitra S. Michaluk P. Pijet B. Stefaniuk M. Kaczmarek L. (2019). MMPs in learning and memory and neuropsychiatric disorders. *Cell. Mol. Life Sci.* 76 3207–3228. 10.1007/s00018-019-03180-8 31172215 PMC6647627

[B12] Bijata M. Labus J. Guseva D. Stawarski M. Butzlaff M. Dzwonek J. (2017). Synaptic remodeling depends on signaling between serotonin receptors and the extracellular matrix. *Cell Rep.* 19 1767–1782. 10.1016/j.celrep.2017.05.023 28564597

[B13] Bradbury E. J. Moon L. D. Popat R. J. King V. R. Bennett G. S. Patel P. N. (2002). Chondroitinase ABC promotes functional recovery after spinal cord injury. *Nature* 416:636. 10.1038/416636a 11948352

[B14] Brakebusch C. Seidenbecher C. I. Asztely F. Rauch U. Matthies H. Meyer H. (2002). Brevican-deficient mice display impaired hippocampal CA1 long-term potentiation but show no obvious deficits in learning and memory. *Mol. Cell. Biol.* 22 7417–7427. 10.1128/MCB.22.21.7417-7427.2002 12370289 PMC135663

[B15] Brittis P. A. Canning D. R. Silver J. (1992). Chondroitin sulfate as a regulator of neuronal patterning in the retina. *Science* 255 733–736. 10.1126/science.1738848 1738848

[B16] Brkic M. Balusu S. Libert C. Vandenbroucke R. E. (2015). Friends or Foes: Matrix metalloproteinases and their multifaceted roles in neurodegenerative diseases. *Mediators Inflamm.* 2015 620581–620581. 10.1155/2015/620581 26538832 PMC4619970

[B17] Broekaart D. W. Bertran A. Jia S. Korotkov A. Senkov O. Bongaarts A. (2021). The matrix metalloproteinase inhibitor IPR-179 has antiseizure and antiepileptogenic effects. *J. Clin. Investig.* 131:e138332. 10.1172/JCI138332 33141761 PMC7773344

[B18] Brückner G. Grosche J. Schmidt S. Härtig W. Margolis R. U. Delpech B. (2000). Postnatal development of perineuronal nets in wild-type mice and in a mutant deficient in tenascin-R. *J. Comp. Neurol.* 428 616–629. 10.1002/1096-9861(20001225)428:4<616::AID-CNE3>3.0.CO;2-K 11077416

[B19] Busch S. A. Silver J. (2007). The role of extracellular matrix in CNS regeneration. *Curr. Opin. Neurobiol.* 17 120–127. 10.1016/j.conb.2006.09.004 17223033

[B20] Cabungcal J.-H. Counotte D. S. Lewis E. M. Tejeda H. A. Piantadosi P. Pollock C. (2014). Juvenile antioxidant treatment prevents adult deficits in a developmental model of schizophrenia. *Neuron* 83 1073–1084. 10.1016/j.neuron.2014.07.028 25132466 PMC4418441

[B21] Cabungcal J.-H. Steullet P. Morishita H. Kraftsik R. Cuenod M. Hensch T. K. (2013). Perineuronal nets protect fast-spiking interneurons against oxidative stress. *Proc. Natl. Acad. Sci. U.S.A.* 110 9130–9135. 10.1073/pnas.1300454110 23671099 PMC3670388

[B22] Cafferty W. B. Yang S. H. Duffy P. J. Li S. Strittmatter S. M. (2007). Functional axonal regeneration through astrocytic scar genetically modified to digest chondroitin sulfate proteoglycans. *J. Neurosci.* 27 2176–2185. 10.1523/JNEUROSCI.5176-06.2007 17329414 PMC2848955

[B23] Carceller H. Gramuntell Y. Klimczak P. Nacher J. (2022). Perineuronal nets: subtle structures with large implications. *Neuroscientist*. 10.1177/10738584221106346 [Epub ahead of print]. 35872660

[B24] Carceller H. Guirado R. Ripolles-Campos E. Teruel-Marti V. Nacher J. (2020). Perineuronal nets regulate the inhibitory perisomatic input onto parvalbumin interneurons and γ activity in the prefrontal cortex. *J. Neurosci.* 40 5008–5018. 10.1523/JNEUROSCI.0291-20.2020 32457072 PMC7314408

[B25] Cargill R. Kohama S. G. Struve J. Su W. Banine F. Witkowski E. (2012). Astrocytes in aged nonhuman primate brain gray matter synthesize excess hyaluronan. *Neurobiol. Aging* 33:e13–e24. 10.1016/j.neurobiolaging.2011.07.006 21872361 PMC3227765

[B26] Carstens K. E. Phillips M. L. Pozzo-Miller L. Weinberg R. J. Dudek S. M. (2016). Perineuronal nets suppress plasticity of excitatory synapses on CA2 pyramidal neurons. *J. Neurosci.* 36 6312–6320. 10.1523/JNEUROSCI.0245-16.2016 27277807 PMC4899529

[B27] Carulli D. Pizzorusso T. Kwok J. C. Putignano E. Poli A. Forostyak S. (2010). Animals lacking link protein have attenuated perineuronal nets and persistent plasticity. *Brain* 133 2331–2347. 10.1093/brain/awq145 20566484

[B28] Celio M. R. (1999). Evolution of the concept of “extracellular matrix’ in the brain. *J. Hist. Neurosci.* 8 186–190. 10.1076/jhin.8.2.186.1832 11624300

[B29] Celio M. R. Spreafico R. De Biasi S. Vitellaro-Zuccarello L. (1998). Perineuronal nets: Past and present. *Trends Neurosci.* 21 510–515. 10.1016/S0166-2236(98)01298-3 9881847

[B30] Chang J. J. Stanfill A. Pourmotabbed T. (2016). The role of matrix metalloproteinase polymorphisms in ischemic stroke. *Int. J. Mol. Sci.* 17:1323. 10.3390/ijms17081323 27529234 PMC5000720

[B31] Chaunsali L. Tewari B. P. Sontheimer H. (2021). Perineuronal net dynamics in the pathophysiology of epilepsy. *Epilepsy Curr.* 21 273–281. 10.1177/15357597211018688 34690566 PMC8512927

[B32] Christopherson K. S. Ullian E. M. Stokes C. C. Mullowney C. E. Hell J. W. Agah A. (2005). Thrombospondins are astrocyte-secreted proteins that promote CNS synaptogenesis. *Cell* 120 421–433. 10.1016/j.cell.2004.12.020 15707899

[B33] Crapser J. D. Arreola M. A. Tsourmas K. I. Green K. N. (2021). Microglia as hackers of the matrix: Sculpting synapses and the extracellular space. *Cell. Mol. Immunol.* 18 2472–2488. 10.1038/s41423-021-00751-3 34413489 PMC8546068

[B34] Crapser J. D. Spangenberg E. E. Barahona R. A. Arreola M. A. Hohsfield L. A. Green K. N. (2020b). Microglia facilitate loss of perineuronal nets in the Alzheimer’s disease brain. *EBioMedicine* 58:102919. 10.1016/j.ebiom.2020.102919 32745992 PMC7399129

[B35] Crapser J. D. Ochaba J. Soni N. Reidling J. C. Thompson L. M. Green K. N. (2020a). Microglial depletion prevents extracellular matrix changes and striatal volume reduction in a model of Huntington’s disease. *Brain* 143 266–288. 10.1093/brain/awz363 31848580 PMC6935750

[B36] Dankovich T. M. Kaushik R. Olsthoorn L. H. M. Petersen G. C. Giro P. E. Kluever V. (2021). Extracellular matrix remodeling through endocytosis and resurfacing of Tenascin-R. *Nat. Commun.* 12:7129. 10.1038/s41467-021-27462-7 34880248 PMC8654841

[B37] Dityatev A. Brückner G. Dityateva G. Grosche J. Kleene R. Schachner M. (2007). Activity-dependent formation and functions of chondroitin sulfate-rich extracellular matrix of perineuronal nets. *Dev. Neurobiol.* 67 570–588. 10.1002/dneu.20361 17443809

[B38] Dubey D. McRae P. A. Rankin-Gee E. K. Baranov E. Wandrey L. Rogers S. (2017). Increased metalloproteinase activity in the hippocampus following status epilepticus. *Epilepsy Res.* 132 50–58. 10.1016/j.eplepsyres.2017.02.021 28292736 PMC6690398

[B39] Dziembowska M. Milek J. Janusz A. Rejmak E. Romanowska E. Gorkiewicz T. (2012). Activity-dependent local translation of matrix metalloproteinase-9. *J. Neurosci.* 32 14538–14547. 10.1523/JNEUROSCI.6028-11.2012 23077039 PMC6621441

[B40] Dzwonek J. Rylski M. Kaczmarek L. (2004). Matrix metalloproteinases and their endogenous inhibitors in neuronal physiology of the adult brain. *FEBS Lett.* 567 129–135. 10.1016/j.febslet.2004.03.070 15165905

[B41] Engelhardt B. (2003). Development of the blood-brain barrier. *Cell Tissue Res.* 314 119–129. 10.1007/s00441-003-0751-z 12955493

[B42] Enwright J. F. Sanapala S. Foglio A. Berry R. Fish K. N. Lewis D. A. (2016). Reduced labeling of parvalbumin neurons and perineuronal nets in the dorsolateral prefrontal cortex of subjects with schizophrenia. *Neuropsychopharmacology* 41 2206–2214. 10.1038/npp.2016.24 26868058 PMC4946056

[B43] Escartin C. Galea E. Lakatos A. O’Callaghan J. P. Petzold G. C. Serrano-Pozo A. (2021). Reactive astrocyte nomenclature, definitions, and future directions. *Nat. Neurosci.* 24 312–325. 10.1038/s41593-020-00783-4 33589835 PMC8007081

[B44] Evers M. R. Salmen B. Bukalo O. Rollenhagen A. Bösl M. R. Morellini F. (2002). Impairment of L-type Ca2+ channel-dependent forms of hippocampal synaptic plasticity in mice deficient in the extracellular matrix glycoprotein tenascin-C. *J. Neurosci.* 22 7177–7194. 10.1523/JNEUROSCI.22-16-07177.2002 12177213 PMC6757873

[B45] Falo M. C. Fillmore H. L. Reeves T. M. Phillips L. L. (2006). Matrix metalloproteinase-3 expression profile differentiates adaptive and maladaptive synaptic plasticity induced by traumatic brain injury. *J. Neurosci. Res.* 84 768–781. 10.1002/jnr.20986 16862547

[B46] Favuzzi E. Marques-Smith A. Deogracias R. Winterflood C. M. Sánchez-Aguilera A. Mantoan L. (2017). Activity-dependent gating of parvalbumin interneuron function by the perineuronal net protein brevican. *Neuron* 95 639–655. 10.1016/j.neuron.2017.06.028 28712654

[B47] Fawcett J. W. Oohashi T. Pizzorusso T. (2019). The roles of perineuronal nets and the perinodal extracellular matrix in neuronal function. *Nat. Rev. Neurosci.* 20 451–465. 10.1038/s41583-019-0196-3 31263252

[B48] Fitch M. T. Silver J. (2008). CNS injury, glial scars, and inflammation: Inhibitory extracellular matrices and regeneration failure. *Exp. Neurol.* 209 294–301. 10.1016/j.expneurol.2007.05.014 17617407 PMC2268907

[B49] Foscarin S. Ponchione D. Pajaj E. Leto K. Gawlak M. Wilczynski G. M. (2011). Experience-dependent plasticity and modulation of growth regulatory molecules at central synapses. *PLoS One* 6:e16666. 10.1371/journal.pone.0016666 21304956 PMC3031615

[B50] Foscarin S. Raha-Chowdhury R. Fawcett J. W. Kwok J. C. F. (2017). Brain ageing changes proteoglycan sulfation, rendering perineuronal nets more inhibitory. *Aging* 9 1607–1622. 10.18632/aging.101256 28657900 PMC5509459

[B51] Frischknecht R. Heine M. Perrais D. Seidenbecher C. I. Choquet D. Gundelfinger E. D. (2009). Brain extracellular matrix affects AMPA receptor lateral mobility and short-term synaptic plasticity. *Nat. Neurosci.* 12 897–904. 10.1038/nn.2338 19483686

[B52] Geissler M. Gottschling C. Aguado A. Rauch U. Wetzel C. H. Hatt H. (2013). Primary hippocampal neurons, which lack four crucial extracellular matrix molecules, display abnormalities of synaptic structure and function and severe deficits in perineuronal net formation. *J. Neurosci.* 33 7742–7755. 10.1523/JNEUROSCI.3275-12.2013 23637166 PMC6618965

[B53] George N. Geller H. M. (2018). Extracellular matrix and traumatic brain injury. *J. Neurosci. Res.* 96 573–588. 10.1002/jnr.24151 29344975 PMC5803383

[B54] Giamanco K. A. Matthews R. T. (2012). Deconstructing the perineuronal net: Cellular contributions and molecular composition of the neuronal extracellular matrix. *Neuroscience* 218 367–384. 10.1016/j.neuroscience.2012.05.055 22659016 PMC3400135

[B55] Giamanco K. A. Morawski M. Matthews R. T. (2010). Perineuronal net formation and structure in aggrecan knockout mice. *Neuroscience* 170 1314–1327. 10.1016/j.neuroscience.2010.08.032 20732394

[B56] Gottschall P. E. Howell M. D. (2015). ADAMTS expression and function in central nervous system injury and disorders. *Matrix Biol.* 44 70–76. 10.1016/j.matbio.2015.01.014 25622912 PMC5068130

[B57] Gottschling C. Wegrzyn D. Denecke B. Faissner A. (2019). Elimination of the four extracellular matrix molecules tenascin-C, tenascin-R, brevican and neurocan alters the ratio of excitatory and inhibitory synapses. *Sci. Rep.* 9:13939. 10.1038/s41598-019-50404-9 31558805 PMC6763627

[B58] Guimarães A. Zaremba S. Hockfield S. (1990). Molecular and morphological changes in the cat lateral geniculate nucleus and visual cortex induced by visual deprivation are revealed by monoclonal antibodies Cat-304 and Cat-301. *J. Neurosci.* 10 3014–3024. 10.1523/JNEUROSCI.10-09-03014.1990 1697900 PMC6570239

[B59] Halfter W. Dong S. Yip Y. P. Willem M. Mayer U. (2002). A critical function of the pial basement membrane in cortical histogenesis. *J. Neurosci.* 22 6029–6040. 10.1523/JNEUROSCI.22-14-06029.2002 12122064 PMC6757907

[B60] Harkness J. H. Gonzalez A. E. Bushana P. N. Jorgensen E. T. Hegarty D. M. Di Nardo A. A. (2021). Diurnal changes in perineuronal nets and parvalbumin neurons in the rat medial prefrontal cortex. *Brain Struct. Funct.* 226 1135–1153. 10.1007/s00429-021-02229-4 33585984 PMC8086998

[B61] Härtig W. Derouiche A. Welt K. Brauer K. Grosche J. Mäder M. (1999). Cortical neurons immunoreactive for the potassium channel Kv3. 1b subunit are predominantly surrounded by perineuronal nets presumed as a buffering system for cations. *Brain Res.* 842 15–29. 10.1016/S0006-8993(99)01784-9 10526091

[B62] Hatcher A. Yu K. Meyer J. Aiba I. Deneen B. Noebels J. L. (2020). Pathogenesis of peritumoral hyperexcitability in an immunocompetent CRISPR-based glioblastoma model. *J. Clin. Investig.* 130 2286–2300. 10.1172/JCI133316 32250339 PMC7190940

[B63] Hayani H. Song I. Dityatev A. (2018). Increased excitability and reduced excitatory synaptic input into fast-spiking CA2 interneurons after enzymatic attenuation of extracellular matrix. *Front. Cell. Neurosci.* 12:149. 10.3389/fncel.2018.00149 29899690 PMC5988902

[B64] Hayashi M. K. Nishioka T. Shimizu H. Takahashi K. Kakegawa W. Mikami T. (2019). Hyaluronan synthesis supports glutamate transporter activity. *J. Neurochem.* 150 249–263. 10.1111/jnc.14791 31188471

[B65] Horii-Hayashi N. Sasagawa T. Matsunaga W. Nishi M. (2015). Development and structural variety of the chondroitin sulfate proteoglycans-contained extracellular matrix in the mouse brain. *Neural Plast.* 2015:256389. 10.1155/2015/256389 26649203 PMC4663360

[B66] Hrabetova S. Cognet L. Rusakov D. A. Nägerl U. V. (2018). Unveiling the extracellular space of the brain: From super-resolved microstructure to *in vivo* function. *J. Neurosci.* 38 9355–9363. 10.1523/JNEUROSCI.1664-18.2018 30381427 PMC6706003

[B67] Huang W. C. Kuo W. C. Cherng J. H. Hsu S. H. Chen P. R. Huang S. H. (2006). Chondroitinase ABC promotes axonal re-growth and behavior recovery in spinal cord injury. *Biochem. Biophys. Res. Commun.* 349 963–968. 10.1016/j.bbrc.2006.08.136 16965762

[B68] Hubel D. H. Wiesel T. N. (1965). Binocular interaction in striate cortex of kittens reared with artificial squint. *J. Neurophysiol.* 28 1041–1059. 10.1152/jn.1965.28.6.1041 5883731

[B69] Hubel D. H. Wiesel T. N. (1970). The period of susceptibility to the physiological effects of unilateral eye closure in kittens. *J. Physiol.* 206 419–436. 10.1113/jphysiol.1970.sp009022 5498493 PMC1348655

[B70] Irintchev A. Rollenhagen A. Troncoso E. Kiss J. Z. Schachner M. (2005). Structural and functional aberrations in the cerebral cortex of tenascin-C deficient mice. *Cereb. Cortex* 15 950–962. 10.1093/cercor/bhh195 15537675

[B71] Irvine S. F. Kwok J. C. (2018). Perineuronal nets in spinal motoneurones: Chondroitin sulphate proteoglycan around alpha motoneurones. *Int. J. Mol. Sci.* 19:1172. 10.3390/ijms19041172 29649136 PMC5979458

[B72] Jäger C. Lendvai D. Seeger G. Brückner G. Matthews R. T. Arendt T. (2013). Perineuronal and perisynaptic extracellular matrix in the human spinal cord. *Neuroscience* 238 168–184. 10.1016/j.neuroscience.2013.02.014 23428622

[B73] Jeanne M. Jorgensen J. Gould D. B. (2015). Molecular and genetic analyses of collagen type iv mutant mouse models of spontaneous intracerebral hemorrhage identify mechanisms for stroke prevention. *Circulation* 131 1555–1565. 10.1161/CIRCULATIONAHA.114.013395 25753534 PMC4497509

[B74] Jiang D. Liang J. Noble P. W. (2011). Hyaluronan as an immune regulator in human diseases. *Physiol. Rev.* 91 221–264. 10.1152/physrev.00052.2009 21248167 PMC3051404

[B75] Jones E. V. Bouvier D. S. (2014). Astrocyte-secreted matricellular proteins in CNS remodelling during development and disease. *Neural Plast.* 12:321209. 10.1155/2014/321209 24551460 PMC3914553

[B76] Kamat P. K. Swarnkar S. Rai S. Kumar V. Tyagi N. (2014). Astrocyte mediated MMP-9 activation in the synapse dysfunction: An implication in Alzheimer disease. *Ther. Targets Neurol. Dis.* 1:e243. 10.14800/ttnd.243 25590048 PMC4290019

[B77] Karus M. Denecke B. Ffrench-Constant C. Wiese S. Faissner A. (2011). The extracellular matrix molecule tenascin C modulates expression levels and territories of key patterning genes during spinal cord astrocyte specification. *Development* 138 5321–5331. 10.1242/dev.067413 22071102

[B78] Kelwick R. Desanlis I. Wheeler G. N. Edwards D. R. (2015). The ADAMTS (A disintegrin and metalloproteinase with thrombospondin motifs) family. *Genome Biol.* 16:113. 10.1186/s13059-015-0676-3 26025392 PMC4448532

[B79] Khodaie-Ardakani M.-R. Mirshafiee O. Farokhnia M. Tajdini M. Modabbernia A. Rezaei F. (2014). Minocycline add-on to risperidone for treatment of negative symptoms in patients with stable schizophrenia: Randomized double-blind placebo-controlled study. *Psychiatry Res.* 215 540–546. 10.1016/j.psychres.2013.12.051 24480077

[B80] Kim E. M. Hwang O. (2011). Role of matrix metalloproteinase-3 in neurodegeneration. *J. Neurochem.* 116 22–32. 10.1111/j.1471-4159.2010.07082.x 21044079

[B81] Kim S. Y. Porter B. E. Friedman A. Kaufer D. (2016). A potential role for glia-derived extracellular matrix remodeling in postinjury epilepsy. *J. Neurosci. Res.* 94 794–803. 10.1002/jnr.23758 27265805

[B82] Kim S. Y. Senatorov V. V. Morrissey C. S. Lippmann K. Vazquez O. Milikovsky D. Z. (2017). β signaling is associated with changes in inflammatory gene expression and perineuronal net degradation around inhibitory neurons following various neurological insults. *Sci. Rep.* 7:7711. 10.1038/s41598-017-07394-3 28794441 PMC5550510

[B83] Kochlamazashvili G. Henneberger C. Bukalo O. Dvoretskova E. Senkov O. Lievens P. M.-J. (2010). The extracellular matrix molecule hyaluronic acid regulates hippocampal synaptic plasticity by modulating postsynaptic L-type Ca 2+ channels. *Neuron* 67 116–128. 10.1016/j.neuron.2010.05.030 20624596 PMC3378029

[B84] Korotchenko S. Zanacchi F. C. Diaspro A. Dityatev A. (2014). “Zooming in on the (Peri)synaptic Extracellular Matrix,” in *Nanoscale Imaging of Synapses: New Concepts and Opportunities*, eds Nägerl U. V. Triller A. (Berlin: Springer), 187–203. 10.1007/978-1-4614-9179-8_10

[B85] Kucukdereli H. Allen N. J. Lee A. T. Feng A. Ozlu M. I. Conatser L. M. (2011). Control of excitatory CNS synaptogenesis by astrocyte-secreted proteins Hevin and SPARC. *Proc. Natl. Acad. Sci. U.S.A.* 108:E440–E449. 10.1073/pnas.1104977108 21788491 PMC3156217

[B86] Kwok J. C. Dick G. Wang D. Fawcett J. W. (2011). Extracellular matrix and perineuronal nets in CNS repair. *Dev. Neurobiol.* 71 1073–1089. 10.1002/dneu.20974 21898855

[B87] Laabs T. Carulli D. Geller H. M. Fawcett J. W. (2005). Chondroitin sulfate proteoglycans in neural development and regeneration. *Curr. Opin. Neurobiol.* 15 116–120. 10.1016/j.conb.2005.01.014 15721753

[B88] Lander C. Kind P. Maleski M. Hockfield S. (1997). A family of activity-dependent neuronal cell-surface chondroitin sulfate proteoglycans in cat visual cortex. *J. Neurosci.* 17 1928–1939. 10.1523/JNEUROSCI.17-06-01928.1997 9045722 PMC6793771

[B89] Lasek A. W. Chen H. Chen W. Y. (2018). Releasing addiction memories trapped in perineuronal nets. *Trends Genet.* 34 197–208. 10.1016/j.tig.2017.12.004 29289347 PMC5834377

[B90] Lau L. W. Cua R. Keough M. B. Haylock-Jacobs S. Yong V. W. (2013). Pathophysiology of the brain extracellular matrix: A new target for remyelination. *Nat. Rev. Neurosci.* 14:722. 10.1038/nrn3550 23985834

[B91] Lee H. McKeon R. J. Bellamkonda R. V. (2010). Sustained delivery of thermostabilized chABC enhances axonal sprouting and functional recovery after spinal cord injury. *Proc. Natl. Acad. Sci. U.S.A.* 107 3340–3345. 10.1073/pnas.0905437106 19884507 PMC2840440

[B92] Lei Y. Han H. Yuan F. Javeed A. Zhao Y. (2017). The brain interstitial system: Anatomy, modeling, *in vivo* measurement, and applications. *Prog. Neurobiol.* 157 230–246. 10.1016/j.pneurobio.2015.12.007 26837044

[B93] Lensjø K. K. Christensen A. C. Tennøe S. Fyhn M. Hafting T. (2017a). Differential expression and cell-type specificity of perineuronal nets in hippocampus, medial entorhinal cortex, and visual cortex examined in the Rat and Mouse. *eNeuro* 4:ENEURO.0379–16.2017. 10.1523/ENEURO.0379-16.2017 28593193 PMC5461557

[B94] Lensjø K. K. Lepperød M. E. Dick G. Hafting T. Fyhn M. (2017b). Removal of perineuronal nets unlocks juvenile plasticity through network mechanisms of decreased inhibition and increased gamma activity. *J. Neurosci.* 37 1269–1283. 10.1523/JNEUROSCI.2504-16.2016 28039374 PMC6596863

[B95] Levkovitz Y. Mendlovich S. Riwkes S. Braw Y. Levkovitch-Verbin H. Gal G. (2009). A double-blind, randomized study of minocycline for the treatment of negative and cognitive symptoms in early-phase schizophrenia. *J. Clin. Psychiatry* 71 138–149. 10.4088/JCP.08m04666yel 19895780

[B96] Liddelow S. A. Guttenplan K. A. Clarke L. E. Bennett F. C. Bohlen C. J. Schirmer L. (2017). Neurotoxic reactive astrocytes are induced by activated microglia. *Nature* 541:481. 10.1038/nature21029 28099414 PMC5404890

[B97] Lindwall C. Olsson M. Osman A. M. Kuhn H. G. Curtis M. A. (2013). Selective expression of hyaluronan and receptor for hyaluronan mediated motility (Rhamm) in the adult mouse subventricular zone and rostral migratory stream and in ischemic cortex. *Brain Res.* 1503 62–77. 10.1016/j.brainres.2013.01.045 23391595

[B98] Liu Y. J. Spangenberg E. E. Tang B. Holmes T. C. Green K. N. Xu X. (2021). Microglia elimination increases neural circuit connectivity and activity in Adult Mouse cortex. *J. Neurosci.* 41 1274–1287. 10.1523/JNEUROSCI.2140-20.2020 33380470 PMC7888230

[B99] Lorenzo Bozzelli P. Alaiyed S. Kim E. Villapol S. Conant K. (2018). Proteolytic remodeling of perineuronal nets: Effects on synaptic plasticity and neuronal population dynamics. *Neural Plast.* 2018:5735789. 10.1155/2018/5735789 29531525 PMC5817213

[B100] Lu P. Takai K. Weaver V. M. Werb Z. (2011). Extracellular matrix degradation and remodeling in development and disease. *Cold Spring Harb. Perspect. Biol.* 3:a005058. 10.1101/cshperspect.a005058 21917992 PMC3225943

[B101] Mascio G. Notartomaso S. Martinello K. Liberatore F. Bucci D. Imbriglio T. (2022). Progressive build-up of perineuronal nets in the somatosensory cortex is associated with the development of chronic pain in mice. *J. Neurosci.* 42 3037–3048. 10.1523/JNEUROSCI.1714-21.2022 35193928 PMC8985861

[B102] Mauney S. A. Athanas K. M. Pantazopoulos H. Shaskan N. Passeri E. Berretta S. (2013). Developmental pattern of perineuronal nets in the human prefrontal cortex and their deficit in schizophrenia. *Biol. Psychiatry* 74 427–435. 23790226 10.1016/j.biopsych.2013.05.007 PMC3752333

[B103] McKeon R. J. Jurynec M. J. Buck C. R. (1999). The chondroitin sulfate proteoglycans neurocan and phosphacan are expressed by reactive astrocytes in the chronic CNS glial scar. *J. Neurosci.* 19 10778–10788. 10.1523/JNEUROSCI.19-24-10778.1999 10594061 PMC6784959

[B104] McRae P. A. Porter B. E. (2012). The perineuronal net component of the extracellular matrix in plasticity and epilepsy. *Neurochem. Int.* 61 963–972. 10.1016/j.neuint.2012.08.007 22954428 PMC3930202

[B105] McRae P. A. Baranov E. Rogers S. L. Porter B. E. (2012). Persistent decrease in multiple components of the perineuronal net following status epilepticus. *Eur. J. Neurosci.* 36 3471–3482. 22934955 10.1111/j.1460-9568.2012.08268.x PMC4058987

[B106] McRae P. A. Rocco M. M. Kelly G. Brumberg J. C. Matthews R. T. (2007). Sensory deprivation alters aggrecan and perineuronal net expression in the mouse barrel cortex. *J. Neurosci.* 27 5405–5413. 10.1523/JNEUROSCI.5425-06.2007 17507562 PMC6672348

[B107] Mitlöhner J. Kaushik R. Niekisch H. Blondiaux A. Gee C. E. Happel M. F. K. (2020). Dopamine receptor activation modulates the integrity of the perisynaptic extracellular matrix at excitatory synapses. *Cells* 9:260. 10.3390/cells9020260 31972963 PMC7073179

[B108] Miyata S. Kitagawa H. (2016). Chondroitin 6-sulfation regulates perineuronal net formation by controlling the stability of aggrecan. *Neural Plast.* 2016:1305801. 10.1155/2016/1305801 27057358 PMC4738747

[B109] Miyata S. Komatsu Y. Yoshimura Y. Taya C. Kitagawa H. (2012). Persistent cortical plasticity by upregulation of chondroitin 6-sulfation. *Nat. Neurosci.* 15 414–422. 10.1038/nn.3023 22246436

[B110] Miyata S. Nishimura Y. Hayashi N. Oohira A. (2005). Construction of perineuronal net-like structure by cortical neurons in culture. *Neuroscience* 136 95–104. 10.1016/j.neuroscience.2005.07.031 16182457

[B111] Morawski M. Brückner G. Jäger C. Seeger G. Arendt T. (2010). Neurons associated with aggrecan-based perineuronal nets are protected against tau pathology in subcortical regions in Alzheimer’s disease. *Neuroscience* 169 1347–1363. 10.1016/j.neuroscience.2010.05.022 20497908

[B112] Morawski M. Brückner G. Jäger C. Seeger G. Matthews R. T. Arendt T. (2012). Involvement of perineuronal and perisynaptic extracellular matrix in Alzheimer’s disease neuropathology. *Brain Pathol.* 22 547–561. 10.1111/j.1750-3639.2011.00557.x 22126211 PMC3639011

[B113] Morawski M. Dityatev A. Hartlage-Rübsamen M. Blosa M. Holzer M. Flach K. (2014). Tenascin-R promotes assembly of the extracellular matrix of perineuronal nets via clustering of aggrecan. *Philos. Trans. R. Soc. Lond. B Biol. Sci.* 369:20140046. 10.1098/rstb.2014.0046 25225104 PMC4173296

[B114] Morawski M. Reinert T. Meyer-Klaucke W. Wagner F. E. Tröger W. Reinert A. (2015). Ion exchanger in the brain: Quantitative analysis of perineuronally fixed anionic binding sites suggests diffusion barriers with ion sorting properties. *Sci. Rep.* 5:16471. 10.1038/srep16471 26621052 PMC4664884

[B115] Morikawa S. Ikegaya Y. Narita M. Tamura H. (2017). Activation of perineuronal net-expressing excitatory neurons during associative memory encoding and retrieval. *Sci. Rep.* 7:46024. 10.1038/srep46024 28378772 PMC5380958

[B116] Muir E. Adcock K. Morgenstern D. Clayton R. Von Stillfried N. Rhodes K. (2002). Matrix metalloproteases and their inhibitors are produced by overlapping populations of activated astrocytes. *Mol. Brain Res.* 100 103–117. 10.1016/S0169-328X(02)00132-8 12008026

[B117] Nakamura M. Nakano K. Morita S. Nakashima T. Oohira A. Miyata S. (2009). Expression of chondroitin sulfate proteoglycans in barrel field of mouse and rat somatosensory cortex. *Brain Res.* 1252 117–129. 10.1016/j.brainres.2008.11.022 19056358

[B118] Nguyen P. T. Dorman L. C. Pan S. Vainchtein I. D. Han R. T. Nakao-Inoue H. (2020). Microglial remodeling of the extracellular matrix promotes synapse plasticity. *Cell* 182 388–403.e15. 10.1016/j.cell.2020.05.050 32615087 PMC7497728

[B119] Orlando C. Ster J. Gerber U. Fawcett J. W. Raineteau O. (2012). Perisynaptic chondroitin sulfate proteoglycans restrict structural plasticity in an integrin-dependent manner. *J. Neurosci.* 32 18009–18017. 10.1523/JNEUROSCI.2406-12.2012 23238717 PMC6621736

[B120] Pantazopoulos H. Berretta S. (2016). In sickness and in health: Perineuronal nets and synaptic plasticity in psychiatric disorders. *Neural Plast.* 2016:9847696. 10.1155/2016/9847696 26839720 PMC4709762

[B121] Pantazopoulos H. Gisabella B. Rexrode L. Benefield D. Yildiz E. Seltzer P. (2020). Circadian rhythms of perineuronal net composition. *eNeuro* 7:ENEURO.0034–19.2020. 10.1523/ENEURO.0034-19.2020 32719104 PMC7405073

[B122] Pantazopoulos H. Hossain N. M. Chelini G. Durning P. Barbas H. Zikopoulos B. (2022). Chondroitin sulphate proteoglycan axonal coats in the human mediodorsal thalamic nucleus. *Front. Integr. Neurosci.* 16:934764. 10.3389/fnint.2022.934764 35875507 PMC9298528

[B123] Pantazopoulos H. Markota M. Jaquet F. Ghosh D. Wallin A. Santos A. (2015). Aggrecan and chondroitin-6-sulfate abnormalities in schizophrenia and bipolar disorder: A postmortem study on the amygdala. *Transl. Psychiatry* 5:e496–e496. 10.1038/tp.2014.128 25603412 PMC4312825

[B124] Pantazopoulos H. Woo T.-U. W. Lim M. P. Lange N. Berretta S. (2010). Extracellular matrix-glial abnormalities in the amygdala and entorhinal cortex of subjects diagnosed with schizophrenia. *Arch. Gen. Psychiatry* 67 155–166. 10.1001/archgenpsychiatry.2009.196 20124115 PMC4208310

[B125] Patel D. C. Tewari B. P. Chaunsali L. Sontheimer H. (2019). Neuron–glia interactions in the pathophysiology of epilepsy. *Nat. Rev. Neurosci.* 20 282–297. 10.1038/s41583-019-0126-4 30792501 PMC8558781

[B126] Perkins K. L. Arranz A. M. Yamaguchi Y. Hrabetova S. (2017). Brain extracellular space, hyaluronan, and the prevention of epileptic seizures. *Rev. Neurosci.* 28 869–892. 10.1515/revneuro-2017-0017 28779572 PMC5705429

[B127] Peters A. Sherman L. S. (2020). Diverse Roles for hyaluronan and hyaluronan receptors in the developing and adult nervous system. *Int. J. Mol. Sci.* 21:5988. 10.3390/ijms21175988 32825309 PMC7504301

[B128] Phatnani H. Maniatis T. (2015). Astrocytes in neurodegenerative disease. *Cold Spring Harb Perspect. Biol.* 7:a020628. 10.1101/cshperspect.a020628 25877220 PMC4448607

[B129] Pizzorusso T. Medini P. Berardi N. Chierzi S. Fawcett J. W. Maffei L. (2002). Reactivation of ocular dominance plasticity in the adult visual cortex. *Science* 298 1248–1251. 10.1126/science.1072699 12424383

[B130] Powell E. M. Geller H. M. (1999). Dissection of astrocyte-mediated cues in neuronal guidance and process extension. *Glia* 26 73–83. 10.1002/(SICI)1098-1136(199903)26:1<73::AID-GLIA8>3.0.CO;2-S 10088674

[B131] Powell E. M. Fawcett J. W. Geller H. M. (1997). Proteoglycans provide neurite guidance at an astrocyte boundary. *Mol. Cell. Neurosci.* 10 27–42. 9361286 10.1006/mcne.1997.0629

[B132] Pu A. Stephenson E. L. Yong V. W. (2018). The extracellular matrix: focus on oligodendrocyte biology and targeting CSPGs for remyelination therapies. *Glia* 66 1809–1825. 10.1002/glia.23333 29603376

[B133] Quattromani M. J. Pruvost M. Guerreiro C. Backlund F. Englund E. Aspberg A. (2018). Extracellular matrix modulation is driven by experience-dependent plasticity during stroke recovery. *Mol. Neurobiol.* 55 2196–2213. 10.1007/s12035-017-0461-2 28290150 PMC5840227

[B134] Rankin-Gee E. K. McRae P. A. Baranov E. Rogers S. Wandrey L. Porter B. E. (2015). Perineuronal net degradation in epilepsy. *Epilepsia* 56 1124–1133. 10.1111/epi.13026 26032766

[B135] Rauch U. (2007). Brain matrix: Structure, turnover and necessity. *Biochem. Soc. Trans.* 35 656–660. 10.1042/BST0350656 17635114

[B136] Reed M. J. Damodarasamy M. Banks W. A. (2019). The extracellular matrix of the blood–brain barrier: Structural and functional roles in health, aging, and Alzheimer’s disease. *Tissue Barriers* 7:1651157. 10.1080/21688370.2019.1651157 31505997 PMC6866683

[B137] Reh R. K. Dias B. G. Nelson C. A. III Kaufer D. Werker J. F. Kolb B. (2020). Critical period regulation across multiple timescales. *Proc. Natl. Acad. Sci. U.S.A.* 117 23242–23251. 10.1073/pnas.1820836117 32503914 PMC7519216

[B138] Reichelt A. C. Hare D. J. Bussey T. J. Saksida L. M. (2019). Perineuronal nets: Plasticity, protection, and therapeutic potential. *Trends Neurosci.* 42 458–470. 10.1016/j.tins.2019.04.003 31174916

[B139] Rempe R. G. Hartz A. M. S. Bauer B. (2016). Matrix metalloproteinases in the brain and blood-brain barrier: Versatile breakers and makers. *J. Cereb. Blood Flow Metab.* 36 1481–1507. 10.1177/0271678X16655551 27323783 PMC5012524

[B140] Rempe R. G. Hartz A. M. S. Soldner E. L. B. Sokola B. S. Alluri S. R. Abner E. L. (2018). Matrix metalloproteinase-mediated blood-brain barrier dysfunction in epilepsy. *J. Neurosci.* 38 4301–4315. 10.1523/JNEUROSCI.2751-17.2018 29632167 PMC5932641

[B141] Rhodes K. E. Moon L. D. F. Fawcett J. W. (2003). Inhibiting cell proliferation during formation of the glial scar: Effects on axon regeneration in the CNS. *Neuroscience* 120 41–56. 10.1016/S0306-4522(03)00285-9 12849739

[B142] Ribot J. Breton R. Calvo C.-F. Moulard J. Ezan P. Zapata J. (2021). Astrocytes close the mouse critical period for visual plasticity. *Science* 373 77–81. 10.1126/science.abf5273 34210880

[B143] Rogers S. L. Rankin-Gee E. Risbud R. M. Porter B. E. Marsh E. D. (2018). Normal development of the perineuronal net in humans; in patients with and without epilepsy. *Neuroscience* 384 350–360. 10.1016/j.neuroscience.2018.05.039 29885523 PMC6062204

[B144] Rosenberg G. A. (2002). Matrix metalloproteinases in neuroinflammation. *Glia* 39 279–291. 10.1002/glia.10108 12203394

[B145] Rowlands D. Lensjø K. K. Dinh T. Yang S. Andrews M. R. Hafting T. (2018). Aggrecan directs extracellular matrix-mediated neuronal plasticity. *J. Neurosci.* 38 10102–10113. 10.1523/JNEUROSCI.1122-18.2018 30282728 PMC6596198

[B146] Sherman L. S. Matsumoto S. Su W. Srivastava T. Back S. A. (2015). Hyaluronan synthesis, catabolism, and signaling in neurodegenerative diseases. *Int. J. Cell Biol.* 2015:368584. 10.1155/2015/368584 26448752 PMC4581574

[B147] Silver J. Miller J. H. (2004). Regeneration beyond the glial scar. *Nat. Rev. Neurosci.* 5:146. 10.1038/nrn1326 14735117

[B148] Somaiya R. D. Huebschman N. A. Chaunsali L. Sabbagh U. Carrillo G. L. Tewari B. P. (2022). Development of astrocyte morphology and function in mouse visual thalamus. *J. Comp. Neurol.* 530 945–962. 10.1002/cne.25261 34636034 PMC8957486

[B149] Song I. Dityatev A. (2017). Crosstalk between glia, extracellular matrix and neurons. *Brain Res. Bull.* 136 101–108. 10.1016/j.brainresbull.2017.03.003 28284900

[B150] Strackeljan L. Baczynska E. Cangalaya C. Baidoe-Ansah D. Wlodarczyk J. Kaushik R. (2021). Microglia depletion-induced remodeling of extracellular matrix and excitatory synapses in the hippocampus of adult mice. *Cells* 10:1862. 10.3390/cells10081862 PMC8393852 34440631

[B151] Struve J. Maher P. C. Li Y. Q. Kinney S. Fehlings M. G. Kuntz C. (2005). Disruption of the hyaluronan-based extracellular matrix in spinal cord promotes astrocyte proliferation. *Glia* 52 16–24. 10.1002/glia.20215 15892130

[B152] Sun Z. Y. Bozzelli P. L. Caccavano A. Allen M. Balmuth J. Vicini S. (2018). Disruption of perineuronal nets increases the frequency of sharp wave ripple events. *Hippocampus* 28 42–52. 10.1002/hipo.22804 28921856 PMC6047756

[B153] Susuki K. Chang K. J. Zollinger D. R. Liu Y. Ogawa Y. Eshed-Eisenbach Y. (2013). Three mechanisms assemble central nervous system nodes of Ranvier. *Neuron* 78 469–482. 10.1016/j.neuron.2013.03.005 23664614 PMC3756512

[B154] Suttkus A. Rohn S. Weigel S. Glöckner P. Arendt T. Morawski M. (2014). Aggrecan, link protein and tenascin-R are essential components of the perineuronal net to protect neurons against iron-induced oxidative stress. *Cell Death Dis.* 5:e1119–e1119. 10.1038/cddis.2014.25 24625978 PMC3973247

[B155] Tansley S. Gu N. Guzmán A. U. Cai W. Wong C. Lister K. C. (2022). Microglia-mediated degradation of perineuronal nets promotes pain. *Science* 377 80–86. 10.1126/science.abl6773 35617374

[B156] Testa D. Prochiantz A. Di Nardo A. A. (2019). Perineuronal nets in brain physiology and disease. *Semin. Cell Dev. Biol.* 89 125–135. 10.1016/j.semcdb.2018.09.011 30273653

[B157] Tewari B. P. Sontheimer H. (2019). Protocol to quantitatively assess the structural integrity of perineuronal nets ex vivo. *Bio-protocol* 9:e3234–e3234. 10.21769/BioProtoc.3234 33654764 PMC7854210

[B158] Tewari B. P. Chaunsali L. Campbell S. L. Patel D. C. Goode A. E. Sontheimer H. (2018). Perineuronal nets decrease membrane capacitance of peritumoral fast spiking interneurons in a model of epilepsy. *Nat. Commun.* 9:4724. 10.1038/s41467-018-07113-0 30413686 PMC6226462

[B159] Thomsen M. S. Routhe L. J. Moos T. (2017). The vascular basement membrane in the healthy and pathological brain. *J. Cereb. Blood Flow Metab.* 37 3300–3317. 10.1177/0271678X17722436 28753105 PMC5624399

[B160] Ulbrich P. Khoshneviszadeh M. Jandke S. Schreiber S. Dityatev A. (2021). Interplay between perivascular and perineuronal extracellular matrix remodelling in neurological and psychiatric diseases. *Eur. J. Neurosci.* 53, 3811–3830. 10.1111/ejn.14887 32594588

[B161] Uriarte N. Ferreño M. Méndez D. Nogueira J. (2020). Reorganization of perineuronal nets in the medial preoptic Area during the reproductive cycle in female Rats. *Sci. Rep.* 10:5479. 10.1038/s41598-020-62163-z 32214157 PMC7096482

[B162] Vainchtein I. D. Chin G. Cho F. S. Kelley K. W. Miller J. G. Chien E. C. (2018). Astrocyte-derived interleukin-33 promotes microglial synapse engulfment and neural circuit development. *Science* 359 1269–1273. 10.1126/science.aal3589 29420261 PMC6070131

[B163] Van Hove I. Lemmens K. Van de Velde S. Verslegers M. Moons L. (2012). Matrix metalloproteinase-3 in the central nervous system: A look on the bright side. *J. Neurochem.* 123 203–216. 10.1111/j.1471-4159.2012.07900.x 22862420

[B164] van’t Spijker H. M. Kwok J. C. (2017). A sweet talk: The molecular systems of perineuronal nets in controlling neuronal communication. *Front. Integr. Neurosci.* 11:33. 10.3389/fnint.2017.00033 29249944 PMC5717013

[B165] Venturino A. Schulz R. De Jesús-Cortés H. Maes M. E. Nagy B. Reilly-Andújar F. (2021). Microglia enable mature perineuronal nets disassembly upon anesthetic ketamine exposure or 60-Hz light entrainment in the healthy brain. *Cell Rep.* 36:109313. 10.1016/j.celrep.2021.109313 34233180 PMC8284881

[B166] Verslegers M. Lemmens K. Van Hove I. Moons L. (2013). Matrix metalloproteinase-2 and -9 as promising benefactors in development, plasticity and repair of the nervous system. *Prog. Neurobiol.* 105 60–78. 23567503 10.1016/j.pneurobio.2013.03.004

[B167] Wang D. Fawcett J. (2012). The perineuronal net and the control of CNS plasticity. *Cell Tissue Res.* 349 147–160. 10.1007/s00441-012-1375-y 22437874

[B168] Weber P. Bartsch U. Rasband M. N. Czaniera R. Lang Y. Bluethmann H. (1999). Mice deficient for tenascin-R display alterations of the extracellular matrix and decreased axonal conduction velocities in the CNS. *J. Neurosci.* 19 4245–4262. 10.1523/JNEUROSCI.19-11-04245.1999 10341229 PMC6782606

[B169] Wen T. H. Binder D. K. Ethell I. M. Razak K. A. (2018b). The perineuronal ‘safety’net? Perineuronal net abnormalities in neurological disorders. *Front. Mol. Neurosci.* 11:270. 10.3389/fnmol.2018.00270 30123106 PMC6085424

[B170] Wen T. H. Afroz S. Reinhard S. M. Palacios A. R. Tapia K. Binder D. K. (2018a). Genetic reduction of matrix metalloproteinase-9 promotes formation of perineuronal nets around parvalbumin-expressing interneurons and normalizes auditory cortex responses in developing Fmr1 knock-out mice. *Cereb. Cortex* 28 3951–3964. 10.1093/cercor/bhx258 29040407 PMC6188540

[B171] Wiese S. Karus M. Faissner A. (2012). Astrocytes as a source for extracellular matrix molecules and cytokines. *Front. Pharmacol.* 3:120. 10.3389/fphar.2012.00120 22740833 PMC3382726

[B172] Wingert J. C. Sorg B. A. (2021). Impact of perineuronal nets on electrophysiology of parvalbumin interneurons, principal neurons, and brain oscillations: A review. *Front. Synaptic Neurosci.* 13:673210. 10.3389/fnsyn.2021.673210 34040511 PMC8141737

[B173] Wolf S. A. Boddeke H. W. Kettenmann H. (2017). Microglia in physiology and disease. *Annu. Rev. Physiol.* 79 619–643. 10.1146/annurev-physiol-022516-034406 27959620

[B174] Yao Y. Chen Z. L. Norris E. H. Strickland S. (2014). Astrocytic laminin regulates pericyte differentiation and maintains blood brain barrier integrity. *Nat. Commun.* 5:3413. 10.1038/ncomms4413 24583950 PMC3992931

[B175] Yong V. W. Power C. Forsyth P. Edwards D. R. (2001). Metalloproteinases in biology and pathology of the nervous system. *Nat. Rev. Neurosci.* 2:502. 10.1038/35081571 11433375 PMC7097548

[B176] Yu P. Pearson C. S. Geller H. M. (2018). Flexible roles for proteoglycan sulfation and receptor signaling. *Trends Neurosci.* 41 47–61. 10.1016/j.tins.2017.10.005 29150096 PMC5748001

[B177] Zhou X.-H. Brakebusch C. Matthies H. Oohashi T. Hirsch E. Moser M. (2001). Neurocan is dispensable for brain development. *Mol. Cell. Biol.* 21 5970–5978. 10.1128/MCB.21.17.5970-5978.2001 11486035 PMC87315

